# The Roles of EDA2R in Ageing and Disease

**DOI:** 10.1111/acel.70282

**Published:** 2025-11-04

**Authors:** Gemma Farrington, Lauren Tonge, Tracy Branagan, Sud Sudirman, Chao Fang, Louis Luk, Serkan Kir, Marco Bolis, Ildus I. Ahmetov, Kehinde Ross

**Affiliations:** ^1^ School of Biological and Environmental Sciences Liverpool John Moores University Liverpool UK; ^2^ Institute for Health Research Liverpool John Moores University Liverpool UK; ^3^ School of Pharmacy and Biological Sciences Liverpool John Moores University Liverpool UK; ^4^ School of Computing and Mathematics Liverpool John Moores University Liverpool UK; ^5^ School of Law and Social Justice University of Liverpool Liverpool UK; ^6^ School of Chemistry Cardiff University Cardiff UK; ^7^ Department of Molecular Biology and Genetics Koç University Istanbul Turkey; ^8^ Computational Oncology Unit, Department of Oncology Istituto di Ricerche Farmacologiche ‘Mario Negri’ IRCCS Milano Italy; ^9^ Institute of Oncology Research Bellinzona Switzerland; ^10^ Laboratory of Genetics of Aging and Longevity Kazan State Medical University Kazan Russia; ^11^ Research Institute for Sport and Exercise Sciences Liverpool John Moores University Liverpool UK

**Keywords:** ageing, alopecia, EDA2R, inflammation, muscle wasting, therapeutic treatment

## Abstract

Ageing is a complex biological process driven, in part, by inflammaging. Recent research identifies the ectodysplasin A2 receptor (EDA2R) as a key regulator of inflammaging and a novel biomarker of ageing, with its expression increasing with age across diverse tissues in humans and animal models. Elevated *EDA2R* gene expression is associated with accelerated ageing, cellular senescence, frailty, obesity, acne, radiation response and increased levels of inflammatory, renal, cardiac and vascular biomarkers. Similarly, elevated EDA2R protein levels, a critical component of the proteomic ageing clock, are associated with a wide range of conditions, including cardiovascular diseases, dementia, Parkinson's disease, mood disorders, post‐traumatic stress disorder, various cancers, osteoarthritis, digestive diseases, diabetes, obesity, chronic obstructive pulmonary disease, ear and eye diseases, renal impairment, systemic autoimmune diseases, anaemia, bacterial infections, myositis, frailty, accelerated biological ageing, shorter telomere length, decreased healthspan and longevity, higher all‐cause mortality and overall poor health. Beyond serving as a biomarker, EDA2R actively drives ageing, as its overexpression induces inflammation and tissue damage, whereas its inhibition mitigates these effects. Mechanistically, EDA2R activates non‐canonical and canonical NF‐κB signalling, promoting pro‐inflammatory and catabolic processes that accelerate ageing phenotypes. Genetic variants of *EDA2R* are linked to alopecia, facial ageing, lipid profiles and prostate cancer. This review explores the structure and function of the *EDA2R* gene and protein, its role in tissue‐specific ageing, and its therapeutic potential for multiple diseases. Although specific EDA2R antagonists are not yet available, interventions like calorie restriction, physical activity and specific supplements show promise in lowering EDA2R levels.

## Graphical abstract

Elevated expression of the ectodysplasin A2 receptor (EDA2R) has been linked to ageing and disease. We appraise the roles of EDA2R in these processes, exploring mechanisms of action, biomarker potential and therapeutic relevance of EDA2R in multiple contexts.

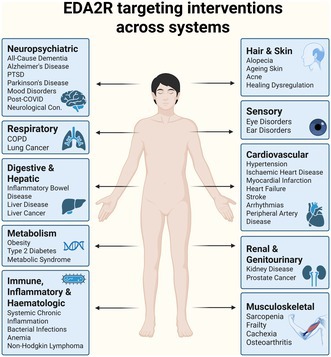

## 1 Introduction

Ageing is a progressive biological process marked by declining physiological and cognitive functions, increasing the risk of cardiovascular, neurodegenerative, metabolic diseases and cancer (Zhang, Ma, et al. 2023; Zhang, Ou, et al. 2023; Guo et al. 2022). Consequently, despite medical advances extending lifespan, ageing populations face rising burdens of chronic disease and disability (Safiri et al. 2023).

Key mechanisms of ageing include cellular senescence, oxidative stress and chronic low‐grade inflammation, collectively termed inflammaging (Baechle et al. 2023). This persistent inflammation arises from intrinsic cellular changes, environmental stressors and immune dysregulation (Halper‐Stromberg and Jabri 2022). Cellular senescence, a state of irreversible cell cycle arrest induced by DNA damage and telomere attrition (Zhang et al. 2022), is a central ageing mechanism. While protective against tumourigenesis, senescent cells accumulate with age and release pro‐inflammatory factors, forming the senescence‐associated secretory phenotype (SASP), which fuels inflammaging (Roger et al. 2021). Oxidative stress, driven by excess reactive oxygen species (ROS) from mitochondrial dysfunction, further promotes senescence and degeneration (Hajam et al. 2022; Afzal et al. 2023). Additional contributors to ageing include genomic instability, telomere shortening, dysregulation of DNA methylation and histone modification, loss of proteostasis, disrupted nutrient sensing, impaired autophagy, stem cell exhaustion, altered intercellular signalling and gut microbiota dysbiosis (Maldonado et al. 2023; Wang et al. 2022; López‐Otín et al. 2023; Ottens et al. 2021). Together, these mechanisms drive functional decline, and disease vulnerability (Iakovou and Kourti 2022).

Inflammaging, characterised by persistent inflammatory mediator elevation without infection, reflects chronic immune activation and impaired resolution (Calder et al. 2017). Identifying molecular regulators of inflammaging is therefore crucial for understanding the biology of ageing and for the development of targeted interventions. One emerging candidate is the ectodysplasin Areceptor (EDA2R), a member of the tumour necrosis factor (TNF) receptor superfamily (Dostert et al. 2019; Wagemann et al. 2025), originally characterised for its role in the embryonic development of ectodermal tissues such as skin and hair follicles (Mikkola 2008; Lee and Tumbar 2012). Mutations in the ectodysplasin A (EDA) gene are associated with X‐linked hypohidrotic ectodermal dysplasia (HED), a genetic disorder characterised by abnormalities in the formation of skin, hair, nails, teeth and sweat glands (Deshmukh and Prashanth 2012; Katthika and Auerkari 2018). The *EDA* gene produces multiple isoforms via alternative splicing, with EDA‐A1 binding EDA receptor (EDAR) and EDA‐A2 binding EDA2R (Rietmann et al. 2024).

The physiological role of EDA‐A2–EDA2R signalling is not fully understood but recent research links EDA2R to inflammation, apoptosis and immune responses, an ageing (Bilgic et al. 2023; von Renesse and Mirtschink 2023; Barbera et al. 2025). There is evidence *EDA2R* may also serve as a biomarker of ageing (Arif et al. 2025). This review, therefore, examines the structure and function of the *EDA2R* gene and protein, evaluates its roles in inflammaging, and explores its involvement in tissue‐specific ageing. Additionally, the review assesses the therapeutic potential of targeting EDA2R to mitigate inflammation, promote healthy ageing and treat conditions such as alopecia, sarcopenia, neurodegeneration and cardiovascular disease.

## 2 *EDA2R* Gene: Structure, Expression and Phenotypic Associations

### 2.1 Genomic Localization and Structure of the *EDA2R* Gene

The *EDA2R* gene (previously known as *XEDAR*, *EDA‐A2R*, *EDAA2R or TNFRSF27*) is located on the X chromosome at cytogenetic band Xq12 and spans 43,662 bases on the reverse strand (chrX:66,595,637–66,639,298; GRCh38/hg38). It has four annotated transcripts in Ensembl (EDA2R‐201, EDA2R‐202, EDA2R‐203 and EDA2R‐204), with the canonical transcript EDA2R‐201 comprising 12 exons and 11 introns (Ensembl 2025).

### 2.2 Tissue‐Specific Expression and Regulation of *EDA2R*

Early studies reported that *EDA2R* is predominantly expressed in ectoderm‐derived tissues such as the skin and hair follicles (Bergqvist et al. 2017). More recent transcriptomic and proteomic data have demonstrated *EDA2R* expression in immune, adipose and cardiovascular tissues, suggesting broader functional roles beyond dermatological processes (Kanoni et al. 2022). In the Genotype‐Tissue Expression (GTEx) Portal (2025), *EDA2R* shows the highest expression in tissues of the reproductive (uterus, cervix, ovary, fallopian tube, prostate and vagina), endocrine (thyroid, adrenal gland and pituitary), vascular (arteries), nervous (tibial nerve) and urinary (bladder) systems, as well as in cultured fibroblasts. Moderate expression is observed in digestive (oesophagus, colon, pancreas, stomach, small intestine), respiratory (lung), exocrine (salivary gland, breast), integumentary (skin), urinary (kidney), reproductive (testis), lymphatic (spleen), muscular (m. gastrocnemius) and adipose tissues. The lowest expression is found in brain tissues, liver and whole blood. The *EDA2R* gene exhibits higher expression in males than in females in adipose tissue, heart, blood vessels, lungs and colon, while its expression is higher in females in the brain and skin (Barbera et al. 2025).

The *EDA2R* gene is located in close genomic proximity to the androgen receptor (*AR*) gene on the X chromosome (Prodi et al. 2008), suggesting potential co‐regulation or functional interaction of these loci, in androgen‐sensitive tissues like the skin and hair follicles (Ceruti et al. 2018). Indeed, data from the GTEx (2025) indicate a positive correlation between *EDA2R* and *AR* gene expression in skeletal muscle in both males and females.

*EDA2R* transcript levels appear to vary with age, developmental cues, hormonal signaling and pathological stimuli, including inflammation (Lan et al. 2020; Barbera et al. 2025). In a comprehensive analysis of the GTEx dataset, Barbera et al. (2025) identified *EDA2R* as a prominent candidate gene whose expression increases with age. This gene showed a strong positive correlation with increasing donor age across all organs examined, consistently ranking among the top hits in both male and female solid tissues and achieving the highest mean correlation coefficient in a comprehensive pan‐tissue analysis. Furthermore, a significant age‐related increase in *EDA2R* expression was observed in tissues from rats and mice suggesting a conserved, species‐independent pattern. These findings highlight the significance of EDA2R in age‐related biological processes across diverse tissues and species.

### 2.3 Genetic Variants in and Near the *EDA2R* Gene: From Pathogenic Mutations to Functional Polymorphisms

Mutations in *EDA2R* have been causally linked to non‐syndromic forms of hypohidrotic ectodermal dysplasia (HED), a condition characterised by abnormalities in hair, sweat glands and teeth (Dorgaleleh et al. 2021). The first documented case of an *EDA2R* mutation involved a 13‐year‐old male with mild HED (Wisniewski and Trzeciak 2012). A hemizygous frameshift mutation in exon 3 of the *EDA2R* gene (c.252delG) was identified, resulting in a premature stop codon 108 nucleotides downstream and producing a truncated, nonfunctional receptor. Clinically, the patient presented with hyperthermia, dry skin, periorbital hyperpigmentation, sparse scalp and facial hair, hypodontia with misshapen teeth, and an absence of sweat gland function confirmed by an iodine test. These findings suggest *EDA2R* modulates skin and hair physiology through both genetic susceptibility and functional mechanisms.

More recently, Henne et al. (2023) investigated the contribution of rare genetic variants to the aetiology of male‐pattern hair loss (MPHL) using exome‐sequencing data from 72,469 male participants in the UK Biobank. The study identified five genes significantly associated with MPHL, including a rare missense variant in the *EDA2R* gene (rs12837393; Pro112Ser), which showed a strong association with the condition (*p* = 3.0 × 10^−12^).

According to the Ensembl database (2025), thousands of genetic variants have been identified within or near the *EDA2R* gene, the majority of which are rare (i.e., have a minor allele frequency of less than 1%). Among these, 16 polymorphisms (i.e., common variants) within the *EDA2R* gene are classified as functional, meaning they influence *EDA2R* gene expression in various tissues, as reported by the GTEx Portal (2025). These include four 3′‐UTR (Untranslated Region) variants (rs11093958, rs12855916, rs1485682 and rs4827380), 11 intronic variants (rs1352015, rs1385696, rs150948525, rs1825564, rs4827499, rs5918658, rs5919161, rs73221526, rs73221528, rs983599 and rs995618) and one missense variant (rs1385699; Arg57Lys). Of these, rs1385699 and rs1352015 have previously been reported to be associated with androgenetic alopecia (Prodi et al. 2008).

Of note, over 1500 polymorphisms, most of which are in linkage disequilibrium, are located near the *EDA2R* gene and influence its expression in various tissues (GTEx Portal 2025). Some of these variants have been associated with ageing‐related and other phenotypes in a pleiotropic manner. For example, the rs1576625 T allele (upstream gene variant 17,262 base pairs away from *EDA2R*) is associated with increased *EDA2R* expression in cultured fibroblasts (*p* = 0.0015) and skin (*p* = 0.0074), as well as with elevated risks of advanced (stage IV) male pattern baldness (*p* = 6.5 × 10^−86^), facial ageing (*p* = 7.3 × 10^−7^) and prostate cancer (*p* = 6.3 × 10^−6^) in the UK Biobank cohort (Open Targets Platform 2025), suggesting that elevated *EDA2R* expression may be an unfavourable trait linked to age‐related and pathological conditions.

Importantly, such intergenic polymorphisms, particularly those located between the *AR* and *EDA2R* genes, may influence the expression of both genes and simultaneously associate with male‐pattern baldness. For example, in the *AR/EDA2R* locus, the rs4827528 demonstrated the strongest association with male‐pattern baldness (*p* < 1 × 10^−350^) in a genome‐wide association study involving over 70,000 men (Pirastu et al. 2017). Consequently, there is ongoing debate as to whether the primary causal gene for baldness is *AR*, *EDA2R*, or whether both contribute independently. To identify the causative gene and elucidate the underlying regulatory mechanisms, fine‐mapping, CRISPR‐based (Clustered Regularly Interspaced Short Palindromic Repeats) functional assays, tissue‐specific eQTL analysis and chromatin conformation studies are necessary.

In a large‐scale trans‐ancestral meta‐analysis of ~450,000 individuals integrating proton nuclear magnetic resonance spectroscopy for 249 circulating metabolites and lipoprotein traits, Zoodsma et al. (2025) identified multiple variants in close proximity to the *EDA2R* gene (rs775361, rs1458818, rs73221602, rs5919209, rs142174325 and rs35176586), which showed significant associations with a diverse spectrum of metabolic phenotypes, including renal marker creatinine; lipid traits such as high‐density lipoprotein (HDL) cholesterol and total triglycerides; structural and compositional parameters of HDL subclasses; and global measures such as total triglycerides and average VLDL (very low‐density lipoprotein) particle diameter, thereby positioning the *EDA2R* locus as a regulator of systemic lipid and lipoprotein metabolism.

Overall, the tissue‐specific expression of *EDA2R* and its genotype–phenotype associations highlight its potential as a biomarker and therapeutic target in dermatological, immunological, metabolic and ageing‐related disorders.

## 3 Structural Biology of the EDA2R Protein

### 3.1 Domain Architecture and Structural Features of EDA2R

The human EDA2R protein, also known as XEDAR, is a 297‐amino‐acid type III transmembrane protein with a molecular mass of 32,759 Da and is a member of the TNFR superfamily (Yan et al. 2000). Like other members of this family, EDA2R contains extracellular cysteine‐rich domains (CRDs) responsible for ligand binding, a transmembrane domain, and a cytoplasmic death or TRAF (TNF receptor‐associated factor)‐binding domain that mediates downstream signalling cascades, including the NF‐κB and JNK (c‐Jun N‐terminal kinase) pathways (Özen and Kir 2024). These signalling events play essential roles in apoptosis, cell survival, inflammation, tissue remodelling and development (Wiens and Glenney 2011).

**FIGURE 1:**
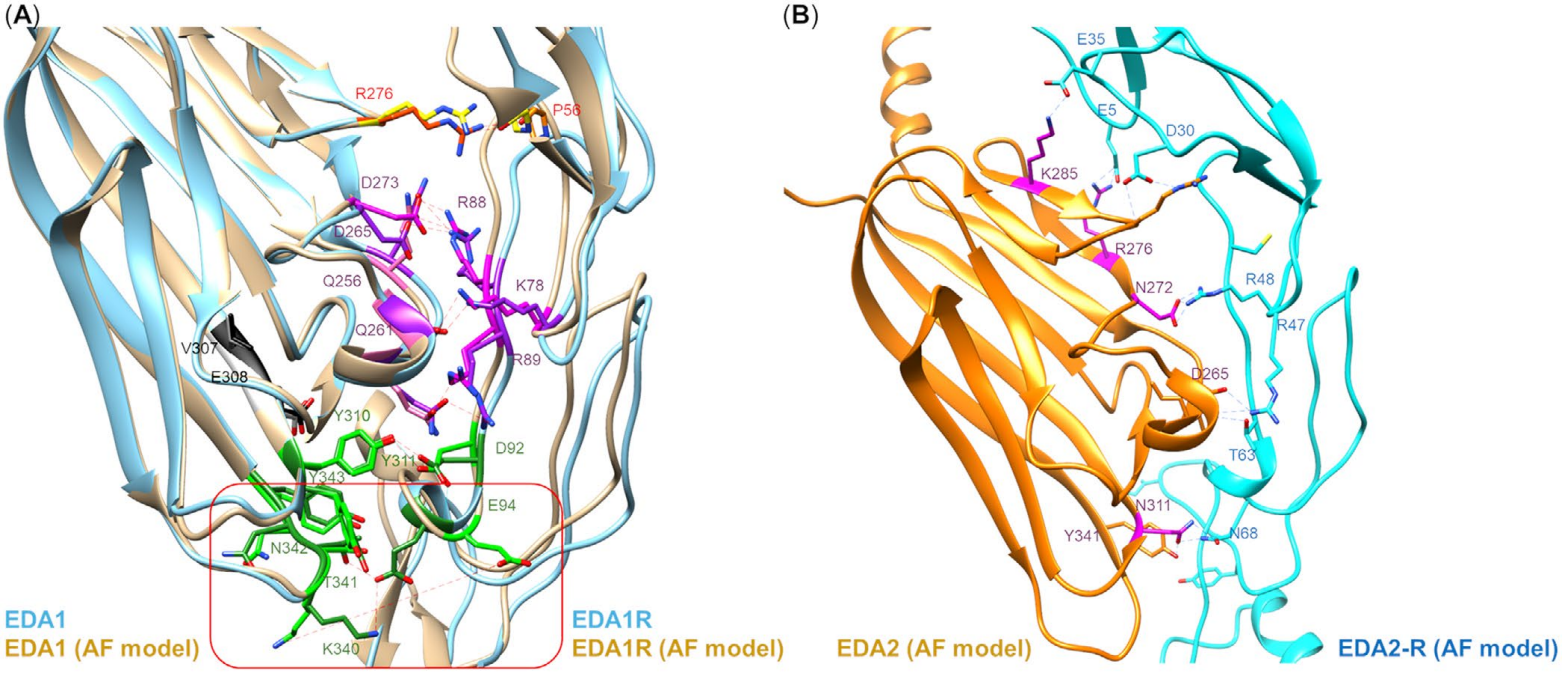
(A) Overlay of crystal structure (Protein Data Bank 7X9G) and AlphaFold 3 (AF3)‐predicted model of EDA1‐EDA1R. Key interactions are indicated with discrepancy highlighted in the red box. (B) AF3‐predicted model of EDA2‐EDA2R with key interactions indicated.

The crystal structure of the EDA‐A2:EDA2R complex has not been reported yet, though the extracellular section of EDA2R and EDA‐A2 have been reported separately (Hymowitz et al. 2003). In contrast, the first high‐resolution crystal structure EDAR bound to its cognate ligand EDA‐A1 was recently reported (Yu et al. 2023). Advances in computational prediction now offer opportunities to explore such interactions in silico. This is relevant for distinguishing EDA‐A1 from EDA‐A2, as the former contains two additional residues (Glu308 and Val309). Nevertheless, our AlphaFold3 (AF3)‐based modelling revealed key discrepancies between predicted and experimentally resolved structures of the EDA‐A1:EDAR complex (Figure 1) Some key hydrogen‐bonding interactions between residues, such as Asp273 and Gln256 of EDA‐A1 with Arg88 of EDAR, and Asp265 with Lys58, were accurately predicted. The interaction between Gln261 and Arg89, as well as residues downstream to Gly308‐Val309 were also captured (Tyr310 and Asp92). However, AF3 failed to predict certain contacts, such as the interaction between Lys340 in EDA‐A1 and Glu94 in EDAR. These findings suggest that while predictive tools provide useful insights, experimental validation remains essential, particularly for flexible, loop‐rich regions. Given that subtle differences in the C‐terminal region (beyond Gly308‐Val309) likely govern selectivity between EDA‐A1 and EDA‐A2, a detailed structural analysis of the EDA‐A2:EDA2R complex will be critical for designing or screening selective binders targeting EDA2R. The AF3‐predicted model of EDA2‐EDA2R (Figure 1B) provides insights into potential interaction sites that can guide such design, but experimental validation is needed.

### 3.2 Predicted or Resolved 3D Structures of EDA2R

Predicting protein 3D structures is vital for drug discovery, protein design and studying variant effects (Ferreira et al. 2015; Huang et al. 2016). Traditional experimental methods such as X‐ray crystallography, nuclear magnetic resonance and cryo‐electron microscopy are accurate but slow and expensive, with fewer than 200,000 structures determined (Burley et al. 2017, 2022).

AI‐based models now enable rapid prediction. Google DeepMind's AlphaFold (AF) series predict protein structures directly from amino acid sequences (Jumper et al. 2021; Abramson et al. 2024). AlphaFold2 integrates database search, evolutionary information (Evoformer) and 3D modelling (Structure module) to iteratively refine protein conformations. Over 200 million predicted structures are freely available (Google DeepMind and EMBL‐EBI 2025). RoseTTAFold (Baek et al. 2021), from the University of Washington, follows a similar principle but uses a lighter, modular architecture that processes sequence, pair, and 3D data simultaneously. It is less computationally demanding but slightly less accurate.

Both models are open source: AF2 under a non‐commercial DeepMind licence, and RoseTTAFold under a BSD‐style licence allowing modification and commercial use. AlphaFold2 achieved near‐experimental accuracy in CASP14, with Global Distance Test (GDT), Template Modelling (TM) score and Local Distance Difference Test (LDDT) values above 0.9 (Heo et al. 2021; Jumper et al. 2021; Zemla 2003; Zhang and Skolnick 2004; Mariani et al. 2013). RoseTTAFold reached 0.87–0.89, remaining attractive for faster, lower‐cost analyses.

**FIGURE 2:**
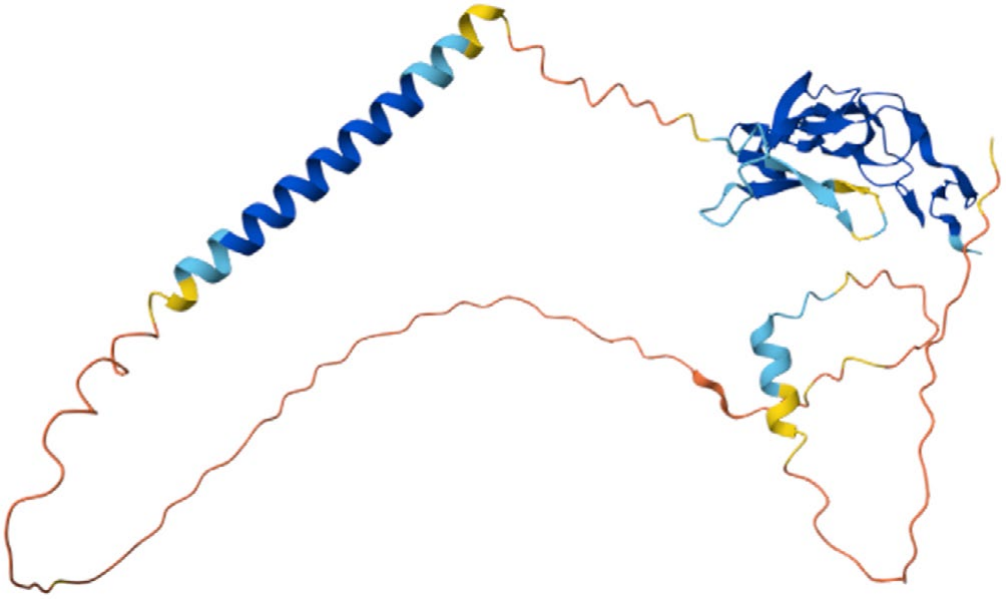
Predicted 3D structure of human EDA2R protein generated by AlphaFold (DeepMind, AlphaFold Protein Structure Database, https://alphafold.ebi.ac.uk/, licensed under CC BY 4.0).

It is worth noting that these AI models were trained using expertly curated and validated 3D structure data obtained using experimental methods. While their performances are very impressive, further improvements on the models will still require results from the more traditional means of protein 3D structure determination. As the experimental 3D structure of the human EDA2R protein remains unresolved, a computational model of EDA2R predicted by AlphaFold is presented in Figure 2 and can be accessed through the AlphaFold Protein Structure Database (Jumper et al. 2021; Varadi et al. 2024), alongside models generated by RoseTTA Fold, via various bioinformatics platforms.

## 4 EDA2R‐Mediated Signalling: Mechanisms, Pathways and Functional Outcomes

Signal transduction by EDAR involves recruitment of the adaptor protein EDARADD (EDAR‐associated via death domain) to engage TRAF proteins, activating downstream signaling cascades. In contrast, the EDA‐A2/EDA2R axis appears to induce nuclear factor‐kappa B (NF‐κB) signaling independently of EDARADD (Sinha et al. 2002).

**FIGURE 3:**
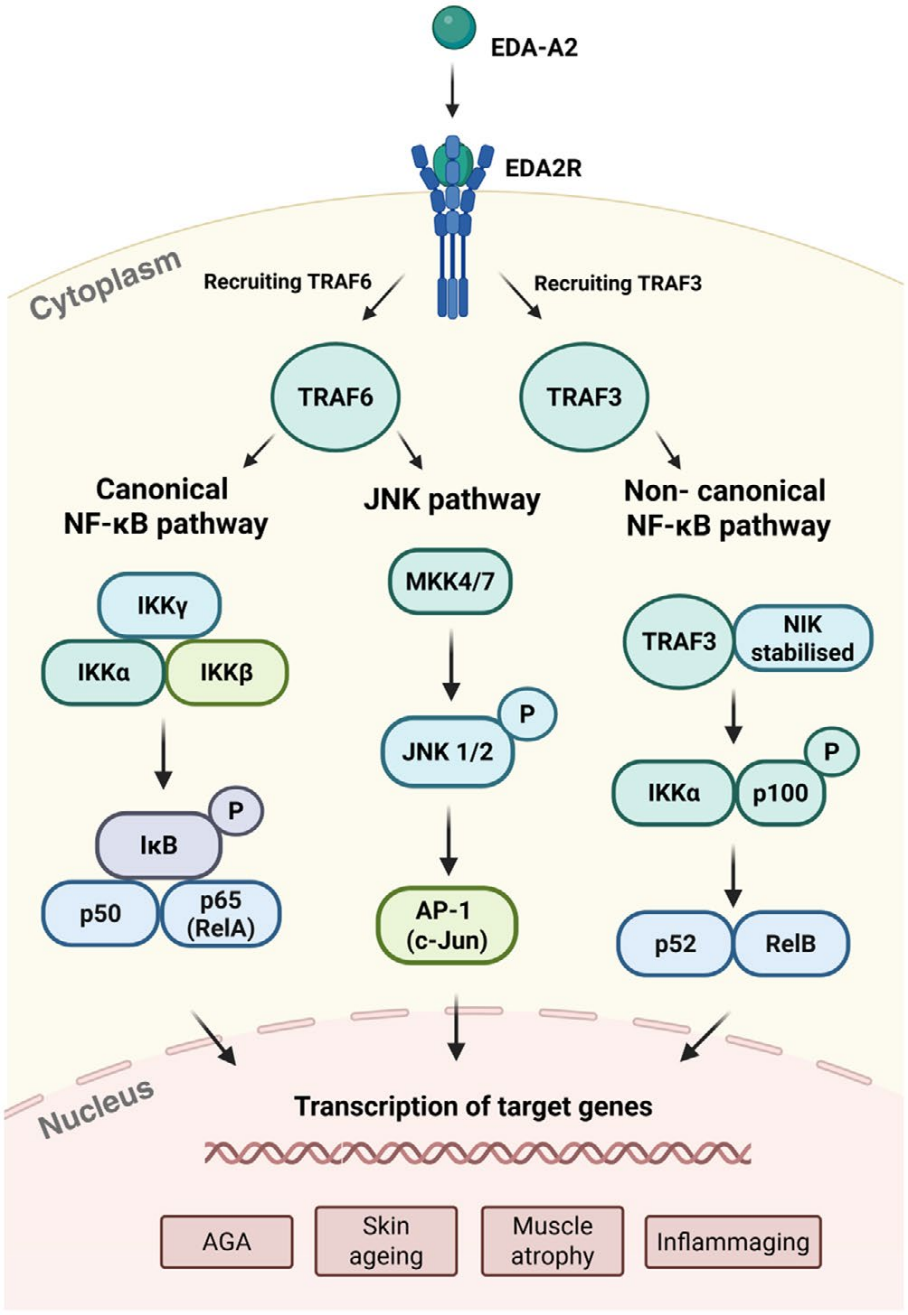
EDA2R‐mediated signalling pathways. The figure illustrates the key signalling pathways activated by EDA2R upon binding with EDA‐A2. Ligand binding induces EDA2R trimerisation and recruits TRAF6 and TRAF3, initiating three distinct pathways: The canonical NF‐κB pathway, the JNK pathway and the non‐canonical NF‐κB pathway. In the canonical NF‐κB pathway, TRAF6 activates the IKK complex, leading to the phosphorylation and degradation of IκB. This releases NF‐κB dimers that translocate to the nucleus and activate gene expression involved in inflammation and immune responses. The JNK pathway is also activated by TRAF6 as it phosphorylates JNK1/2; it activates c‐Jun to form the AP‐1 complex that regulates genes associated with apoptosis and cellular stress. In the non‐canonical NF‐κB pathway, TRAF3 facilitates the degradation of NIK, but upon activation, TRAF3 is degraded, resulting in the stabilisation of NIK. This stabilisation allows NIK to activate IKKα, which phosphorylates p100, converting it into p52. The processed p52 then forms a complex with RelB, which moves to the nucleus to regulate genes critical for immune function. These pathways play essential roles in ectodermal tissue development, immune regulation and the pathogenesis of diseases such as androgenetic alopecia (AGA), skin ageing, muscle atrophy and inflammaging (Illustration made in BioRender).

The EDA2R‐dependent activation of the canonical NF‐κB pathway as well as the JNK pathway, and the non‐canonical NF‐κB pathway, contributes to specific downstream biological effects, spanning transcriptional regulation, apoptosis and immune responses (Yang et al. 2022), as illustrated in Figure 3. For both canonical NF‐κB activation and JNK signalling, the ubiquitin ligase TRAF6 plays a key role. In the case of JNK, TRAF6 activates upstream kinases such as MEKK1, which in turn phosphorylate MKK4 and MKK7, leading to the activation of JNK1 and JNK2 (Wu et al. 2025). Activated JNKs phosphorylate c‐Jun, enabling it to dimerise with c‐Fos and form the activator protein‐1 (AP‐1) transcription factor complex (Kciuk et al. 2022). AP‐1 then translocates to the nucleus, where it regulates genes involved in inflammation, apoptosis, and cellular stress responses (Benitez et al. 2024).

For canonical NF‐κB signalling, recruitment of TRAF6 leads to activation of the IκBα kinase (IKK) complex, which consists of IKKα, IKKβ and NF‐κB Essential Modulator (NEMO). Following phosphorylation and degradation of IκBα, nuclear translocation of NF‐κB dimers, primarily p65/RelA and p50, can proceed, enabling them to drive transcription of genes involved in inflammation, immune responses, and cell survival (Cai et al. 2021; Prescott et al. 2021; Moneva‐Sakelarieva et al. 2025).

Unlike the canonical pathway, the non‐canonical pathway requires IKKα and NF‐κB inducing kinase (NIK), but operates independently of IKKβ and NF‐κB Essential Modulator (NEMO) (Sun 2017). Although EDA2R is well characterised for activating the canonical NF‐κB pathway, emerging evidence suggests that its interaction with TRAF3 may also initiate the non‐canonical pathway by activating NIK and IKKα, leading to p100 processing into p52 and nuclear translocation of the p52/RelB complex (Barnabei et al. 2021; Gao et al. 2023). The p52/RelB complex influences proliferation, inflammation and diseases such as autoimmunity and cancer (Kaltschmidt et al. 2021).

## 5 EDA2R as a Biomarker of Disease Susceptibility and Ageing

Biomarkers are quantifiable indicators of biological states or processes that can be used to assess normal physiology, pathological conditions or responses to therapeutic interventions. They play a central role in precision medicine by enabling early diagnosis, monitoring disease progression, predicting treatment response and estimating biological age. Biomarkers encompass gene expression levels, protein concentrations, DNA methylation patterns, circulating metabolites and other measurable features in tissues or biofluids. Each biomarker type offers unique insights: gene expression and epigenetic markers reflect dynamic cellular states; plasma proteins and metabolites serve as systemic indicators of physiological function; and integrated biomarker panels can define molecular clocks or predict disease risk.

In this context, the *EDA2R* gene and its protein product have emerged as promising systemic biomarkers. Mounting evidence implicates EDA2R in broader physiological processes related to ageing, inflammation and multi‐system disease vulnerability. The following section explores the potential of EDA2R as a biomarker by summarising findings from large‐scale proteomic, transcriptomic and functional studies across diverse age‐related and pathological conditions.

### 5.1 *EDA2R* Gene Expression as a Biomarker of Pathophysiological Traits

Utilising large‐scale transcriptomic data from two cohorts, the Netherlands Study of Depression and Anxiety (NESDA, *n* = 2064) and the Netherlands Twin Register (NTR, *n* = 3164), Barbera et al. (2025) independently identified a consistent positive association in both cohorts between *EDA2R* gene expression in blood and elevated levels of circulating C‐reactive protein (CRP), a well‐established biomarker of systemic inflammation, cardiovascular risk and other age‐related pathological conditions (Sproston and Ashworth 2018).

Aryankalayil et al. (2023) examined early molecular responses to whole‐body radiation in mouse liver to identify RNA biomarkers of radiation injury. Transcriptome analysis 48 h post‐irradiation (1–12 Gy) revealed significant changes in mRNA, with *Eda2r* among the top three most upregulated genes, suggesting its potential as a marker of hepatic radiation damage. Supporting these findings in a clinical context, Kaatsch et al. (2025) demonstrated that routine abdominal computed tomography (CT) scans, with doses ranging from 3.75 to 26.95 mGy, induced a dose‐dependent upregulation of *EDA2R* gene expression in peripheral blood cells, as identified through whole transcriptome sequencing, highlighting its role as a biomarker of cellular response to radiation‐induced DNA damage.

EDA2R has also emerged as a significant biomarker in skin‐related disorders, including acne and sebaceous gland dysfunction. A study by Kwack et al. (2024) found that *EDA2R* is overexpressed in the sebaceous glands of acne patients and is directly involved in increasing lipid production. The research showed that EDA2R activation, through EDA‐A2 signalling, enhanced lipid droplet formation in sebocytes and upregulated lipogenic regulators like PPARγ. This mechanism was specific to EDA2R, as interestingly EDA‐A1/EDAR did not show similar effects. These findings highlight the potential of targeting EDA2R in acne treatments to regulate sebum production and reduce inflammation.

In line with these findings on EDA2R's broader biological significance, Barbera et al. (2025) investigated transcriptional changes in mouse models of Hutchinson‐Gilford progeria syndrome (HGPS), a condition widely used to study accelerated ageing. Among the genes analysed, *Eda2r* emerged as one of the most significantly upregulated across various tissues in HGPS mice, with particularly pronounced expression in the aortic artery. This tissue‐specific increase is especially noteworthy, given the critical role of vascular health in ageing and age‐related diseases. These findings highlight EDA2R not only as a contributor to ageing‐associated vascular dysfunction but also as a potential biomarker of accelerated ageing. The study by Barbera et al. (2025) aligns with human genetic data from the UK Biobank cohort, where genetically predicted high expression of *EDA2R* in the skin (based on the rs1576625 polymorphism) has been associated with an increased risk of facial ageing, as reported in the Open Targets Platform (2025). Together, these results from both animal models and human populations consistently support the role of EDA2R in the molecular mechanisms underlying premature and visible ageing.

Arif et al. (2025), analysing data from the SCAPIS‐SciLifeLab wellness profiling longitudinal study, reported that elevated *EDA2R* expression was positively associated with several clinically relevant biomarkers, including higher levels of cystatin C, platelet count, body weight and hip circumference, markers linked to kidney dysfunction and cardiovascular risk (Svensson‐Färbom et al. 2014), chronic inflammation or pro‐thrombotic states (Koupenova et al. 2018) and metabolic risk (Larsson and Burgess 2021). *EDA2R* expression also correlated with troponin T, intima‐media thickness and creatinine, established indicators of myocardial injury (Daubert and Jeremias 2010), vascular ageing (Willeit et al. 2020), and renal impairment (Giavarina et al. 2021), respectively.

In a study by Perez et al. (2022), *EDA2R* expression was significantly upregulated in the vastus lateralis muscle of older adults with frailty compared to healthy older individuals. To further investigate this association, the authors induced cellular senescence in cultured primary human skeletal muscle cells, comprising both myogenic progenitor cells and differentiated myotubes, using the genotoxic agent doxorubicin. Seven days after treatment, quantitative PCR revealed a significant upregulation of *EDA2R* in senescent compared to non‐senescent cells in both cell types. These findings suggest that senescent muscle cells in vitro exhibit molecular features similar to those observed in frail human muscle tissue. The consistent upregulation of *EDA2R* in both models indicates that it may serve as a marker of senescence and suggests its involvement in age‐related muscle decline. In an independent study employing a cachexia model, Bilgic et al. (2023) observed significant upregulation of *Eda2r* in the skeletal muscle of tumour‐bearing mice. Furthermore, *EDA2R* transcript levels were significantly elevated in skeletal muscle biopsies from patients with cachectic lung, colorectal, pancreatic and upper gastrointestinal cancers, as well as in those with Duchenne muscular dystrophy (DMD) and facioscapulohumeral muscular dystrophy (FSHD), conditions characterised by pronounced muscle mass and function loss (Bilgic et al. 2023).

Collectively, the evidence from diverse cohorts, animal models and clinical studies underscores EDA2R gene expression as a robust and versatile biomarker of multiple interconnected pathophysiological processes. This consistent upregulation across tissues and conditions positions EDA2R as a promising molecular target for diagnostic, prognostic and therapeutic strategies in age‐related and degenerative diseases, where its gene expression could be silenced by microRNAs or other epigenetic mechanisms, warranting further longitudinal research to elucidate its mechanistic contributions and clinical translatability.

### 5.2 Association of EDA2R Protein Levels With Pathophysiological Traits

Measuring protein levels, such as EDA2R, provides a more direct insight into physiological processes and disease states than gene expression analysis, as gene expression does not always correspond to protein levels. Importantly, research utilising the UK Biobank's extensive proteomic dataset has consistently identified key biomarkers like EDA2R, with independent analyses of the same data yielding concordant findings, thereby highlighting the robustness of proteomic approaches.

#### 5.2.1 EDA2R as a Systemic Ageing Biomarker

Recent large‐scale proteomic studies have consistently identified EDA2R as one of the most robust biomarkers of biological ageing. Using plasma proteomic data from 45,441 UK Biobank participants, Argentieri et al. (2024) included EDA2R among 204 circulating proteins forming a highly accurate proteomic ageing clock that not only predicted chronological age but also correlated with 18 major chronic diseases, including cardiovascular, metabolic, renal, hepatic, pulmonary, neurodegenerative and oncological conditions, as well as multimorbidity and all‐cause mortality. These associations were independently validated in Chinese (*n* = 3977) and Finnish (*n* = 1990) cohorts, confirming the clock's cross‐population robustness. Notably, EDA2R ranked among the top 20 age‐associated proteins and displayed high connectivity within protein–protein interaction networks, indicating its integrative role across diverse physiological systems.

Consistent with these results, Mörseburg et al. (2025) analysed 1459 circulating proteins in 44,435 UK Biobank participants and similarly identified EDA2R among the top 20 age‐associated proteins, ranking second overall with levels increasing with age. This observation is further supported by Barbera et al. (2025), who identified EDA2R as the top gene with a strong positive correlation between gene expression and donor age across all organs analysed in the GTEx portal.

Adding a sex‐specific perspective, Jin et al. (2025) developed a proteomic‐based ageing clock, ProteAge, constructed separately for males and females. Their analysis revealed that 414 proteins were associated with accelerated ageing in women, compared with 248 in men, with only 148 proteins shared between the sexes. Among these common proteins, EDA2R emerged as one of the most significantly upregulated in both males and females. This result underscores EDA2R as a robust, sex‐independent biomarker linked to an accelerated rate of biological ageing.

Extending this evidence to broader measures of biological ageing, Ma et al. (2025) analysed 51,904 UK Biobank participants to assess associations between 2923 plasma proteins and biological ageing patterns. They found that EDA2R protein levels were positively associated with frailty and several biological age indicators, including PhenoAge, PhenoAge acceleration, KDM‐Biological Age and KDM‐Biological Age acceleration. PhenoAge estimates mortality risk based on clinical biomarkers and chronological age, while KDM‐Biological Age reflects the age at which an individual's physiology matches average biomarker values from the NHANES III cohort. In contrast, EDA2R protein levels were negatively associated with healthspan, parental lifespan, longevity and telomere length in both males and females. Using the same dataset, Kuo et al. (2025) independently identified a significant negative association between EDA2R protein levels and healthspan (*p* = 2.46 × 10^−180^). Collectively, these findings indicate that elevated EDA2R levels are linked to accelerated biological ageing, while inversely related to healthy ageing markers such as healthspan and telomere length. Supporting this conclusion, Wen (2025) reported that EDA2R shows significant positive phenotypic and genetic correlations with proteome‐based biological age gaps in the heart, immune and pulmonary systems, suggesting its role as a multi‐organ ageing biomarker.

In line with these observations, Oh et al. (2025) used plasma proteomics to estimate the biological age of multiple organs in 44,498 UK Biobank participants and evaluate their associations with disease onset and mortality over 17 years. EDA2R emerged as an important marker of organismal‐level ageing, suggesting that higher circulating EDA2R levels may indicate accelerated systemic ageing and increased susceptibility to age‐related pathologies. Interestingly, similar patterns were also found in non‐human primates: Elsworth et al. (2025) reported that EDA2R expression in both cerebrospinal fluid and plasma of monkeys increased with epigenetic age, confirming its evolutionary conservation as an ageing marker.

Collectively, these findings highlight EDA2R as a central molecular marker of systemic ageing and inflammaging, reflecting its potential involvement in maintaining tissue homeostasis and regulating inflammatory responses.

#### 5.2.2 EDA2R and Cardiometabolic, Cardiovascular and Systemic Diseases

Crucially, EDA2R has also been implicated in disease risk. Papier et al. (2024) examined associations between 1463 plasma proteins and the incidence of 19 cancers and nine cancer subsites in UK Biobank participants. They found that elevated levels of EDA2R protein were positively associated with diffuse lymphoma, liver cancer, lung cancer, multiple myeloma, non‐Hodgkin lymphoma, lip and oral cavity cancer, lung adenocarcinoma, lung small cell carcinoma and lung squamous cell carcinoma. Among these, associations with non‐Hodgkin lymphoma and liver cancer remained significant after multiple testing correction stratified by sex.

Further, EDA2R appears to bridge metabolic and systemic diseases. Qian et al. (2025) explored the role of plasma proteins as mediators linking primary cardiometabolic diseases (diabetes, prediabetes and hypertension) to secondary disease development in ∼50,000 UK Biobank participants. Among 395 unique plasma proteins identified across 1461 significant mediation pathways, EDA2R consistently mediated risk from these primary conditions to multiple downstream diseases. Specifically, in individuals with diabetes, EDA2R mediated risks for artery disease, glomerular diseases, cardiac arrest and arrhythmias, heart failure and renal tubulo‐interstitial diseases. Similar mediation was observed in hypertension and prediabetes, indicating EDA2R's function as a central molecular node linking metabolic dysregulation to cardiovascular, renal and respiratory complications.

In addition, Nielsen et al. (2024) identified high plasma levels of EDA2R among the top 10 proteomic predictors of future osteoarthritis in 19,120 UK Biobank participants. Likewise, Vadaq et al. (2023) showed that EDA2R was significantly upregulated in people living with HIV and associated with increased cardiovascular risk. Similarly, Wang et al. (2025) found that plasma EDA2R levels correlated positively with age, smoking, blood pressure, glucose and kidney disease in Chinese adults, highlighting its potential as a marker of cardiometabolic ageing. Consistently, Kuwabara et al. (2025) observed elevated EDA2R in patients with pulmonary arterial hypertension, inversely correlated with cardiac output and exercise capacity.

Interestingly, You et al. (2023) trained a neural network on 52,006 UK Biobank participants to develop a proteomic risk score for disease incidence and mortality, using measurements of 1461 plasma proteins. This integrative score demonstrated strong predictive power, effectively stratifying individual risk for 45 common conditions. Among the top four proteins with broad predictive relevance was EDA2R, which showed significant associations across a wide range of conditions, including bacterial infections, lung and prostate cancers, anaemia, diabetes, obesity, various cardiovascular diseases (hypertension, ischaemic heart disease, arrhythmias, heart failure, stroke and peripheral artery disease), mood disorders, dementia, Parkinson's disease, as well as disorders of the eye, ear, skin, digestive system (including inflammatory bowel disease and liver disease), respiratory system, musculoskeletal system (osteoarthritis) and kidney function (renal failure). In addition, high EDA2R levels were associated with increased risk of all‐cause mortality and cause‐specific mortality related to cancer, nervous, circulatory and respiratory system diseases (You et al. 2023).

In cardiovascular contexts, Chen et al. (2025) identified EDA2R as one of the most broadly associated proteins with incident cardiovascular diseases in 53,026 UK Biobank participants. It was significantly linked to 11 different cardiovascular disease outcomes, including strong associations with heart failure, coronary artery disease, peripheral arterial disease, atrial fibrillation, ischemic stroke, aortic valve stenosis, pulmonary embolism, deep vein thrombosis, abdominal aneurysm, cardiomyopathy and transient ischemic attack. In agreement, Kim et al. (2025) reported that elevated circulating EDA2R levels predicted incident atrial fibrillation. Likewise, Lind et al. (2024) identified EDA2R among the top five proteins most strongly associated with myocardial infarction. Complementing these findings, Guan et al. (2024) demonstrated in animal models of myocardial ischaemia/reperfusion injury that knockdown of *Eda2r* improved cardiac function, reduced oxidative stress and preserved mitochondrial integrity. Together, these studies provide both epidemiological and mechanistic evidence supporting EDA2R as a promising biomarker and potential therapeutic target in cardiovascular disease.

Finally, in the comprehensive pan‐disease blood atlas study by Bueno Álvez et al. (2025), which profiled the circulating proteome of 8262 individuals from biobanks in Sweden, Turkey, Italy, Belgium, Denmark and Norway, EDA2R emerged as the biomarker most strongly associated with age among all analysed proteins. The plasma level of EDA2R showed a dependence on both age and disease status, with age alone explaining 22.5% (*p*.adj = 4.2 × 10^−194^) of the total variability in its concentration, and disease status accounting for an additional 25.3% (*p*.adj = 3.2 × 10^−193^). In particular, compared with healthy controls, patients with various diseases, including acute myeloid leukaemia, alcohol‐related liver disease, chronic liver disease, *E. coli* pyelonephritis, hepatocellular carcinoma, metabolic dysfunction‐associated steatotic liver disease, metastatic melanoma, myeloma, myositis, pancreatic cancer, paediatric kidney tumour, pneumococcal pneumonia, *Staphylococcus aureus* bacteremia, streptococcal soft tissue infection, systemic lupus erythematosus, systemic sclerosis and viral hepatitis–related cirrhosis, showed significantly higher plasma levels of EDA2R.

#### 5.2.3 EDA2R and Neurodegeneration

Multiple studies have implicated EDA2R in cognitive decline and neurodegeneration. Initially, Harris et al. (2020) identified EDA2R as the strongest protein biomarker associated with cognitive decline in older adults (*n* = 5414), with higher circulating levels correlating with lower general fluid cognitive ability and reduced brain volume, suggesting its relevance for age‐related cognitive decline. In a separate study, Llaurador‐Coll et al. (2023), reported that in a cohort of 129 older adults with overweight/obesity and metabolic syndrome, elevated EDA2R levels were linked to lower cognitive performance, indicating its potential as an early marker of cognitive decline.

Further large‐scale analyses strengthened this link: Zhang et al. (2025) and Guo et al. (2024) independently found that elevated EDA2R predicted dementia risk up to 10–15 years before diagnosis, suggesting strong prognostic potential. Zhang et al. (2025), analysing 2923 plasma proteins in 51,296 participants, reported elevated EDA2R levels up to 10–15 years before clinical diagnosis of Alzheimer's disease, suggesting its potential for early detection. Similarly, Guo et al. (2024), analysing 1463 plasma proteins in 52,645 participants without dementia at baseline, identified EDA2R among the top 11 proteins positively associated with incident all‐cause dementia over a 14.1‐year follow‐up.

Gong et al. (2025) reinforced the role of EDA2R in dementia by demonstrating its association with increased risk of all‐cause dementia, including Alzheimer's and vascular dementia. The study investigated associations between 276 proteins and dementia risk among 3249 participants over a median 9.8‐year follow‐up, using a machine learning algorithm to identify the most important predictors of future dementia onset. These findings were subsequently validated in 52,745 individuals from the UK Biobank over a median 13.7‐year follow‐up. Furthermore, Mendelian randomisation analysis provided evidence supporting a potential causal link between circulating EDA2R levels and both Alzheimer's disease and all‐cause dementia (Gong et al. 2025).

Moreover, Kuan et al. (2020) associated elevated EDA2R with comorbid mild cognitive impairment and post‐traumatic stress disorder (PTSD), implying shared neuroinflammatory mechanisms. Supporting its neurological role further, Patel et al. (2021) identified EDA2R as the protein most strongly linked to neuronal injury in COVID‐19 patients, suggesting its involvement in central nervous system damage. Finally, Miclea et al. (2025) found that EDA2R correlated with white matter lesion volume in early multiple sclerosis, pointing to its potential as a biomarker of neuroinflammatory burden.

#### 5.2.4 Translating Evidence on EDA2R Protein Levels Into Clinical Application

Taken together, the findings across the three subsections highlight EDA2R protein's potential as a central biomarker of ageing and disease. To translate these insights into clinical practice, future research should prioritise the validation of EDA2R as a biomarker by: (1) establishing validated reference values for EDA2R expression and protein levels in human blood, stratified by sex and age, with robust cross‐population validation; (2) linking these measurements to actionable clinical insights, such as disease risk stratification, intervention responsiveness and healthspan optimisation, using standardised interpretative frameworks; and (3) ensuring accessibility through cost‐effective assays and scalable analytical pipelines. These steps reflect the Biomarkers of Ageing Consortium's emphasis on biomarkers that are individually robust, clinically meaningful and suitable for widespread application (Biomarkers of Aging Consortium et al. 2024). Anchoring EDA2R validation to these translational principles may support its adoption in both geroscience research and precision health approaches.

## 6 The Role of EDA2R in Muscle Wasting: Sarcopenia and Cachexia

Skeletal muscle plays an important role in producing mechanical force, supporting posture, energy metabolism and body temperature regulation (Feng et al. 2024). Skeletal muscle mass is regulated by a complex network of signalling pathways and gene expression changes, with anabolic processes driven by the mechanistic target of rapamycin (mTOR) pathway, which promotes protein synthesis and ribosomal biogenesis in response to resistance exercise and nutrient availability, and catabolic processes controlled by the ubiquitin proteasome system and the autophagy lysosome pathway, where forkhead box O (FOXO) transcription factors serve as key regulators of muscle protein breakdown (Glass 2003; Schiaffino et al. 2021; Baumert et al. 2024).

Disruption in the balance between anabolic and catabolic processes can lead to skeletal muscle atrophy, which is characterised by excessive protein catabolism resulting in the loss of muscle mass, quality and strength (Jun et al. 2023). This muscle loss can occur as a result of ageing (known as sarcopenia), muscular dystrophies and cachexia. Sarcopenia is primarily caused by ageing‐related factors such as hormonal changes, physical inactivity, chronic inflammation, oxidative stress, mitochondrial dysfunction, poor nutrition, chronic diseases, medication side effects and socioeconomic limitations (Wiedmer et al. 2021; Kiss et al. 2024; Yang, Kua, and Lim 2025; Yang, Jin, et al. 2025), with some individuals being more vulnerable due to their genetic profile (Semenova et al. 2023; Ginevičienė et al. 2025). Muscular dystrophies are rare genetic disorders caused by mutations in over 40 genes essential for muscle structure and function, leading to progressive muscle degeneration, and potential cardiac and respiratory complications (Mercuri et al. 2019). Cachexia is associated with chronic illnesses like cancer (Moreira‐Pais et al. 2021), and is particularly common in individuals with lung, stomach, pancreatic and colorectal cancers (Mariean et al. 2023). There are currently no effective treatments for reversing muscle wasting and therefore cachexia remains a medical challenge (Von Haehling et al. 2017).

Recent studies converge on a potential role for EDA2R signaling in muscle pathophysiology (Perez et al. 2022; Bilgic et al. 2023; Özen and Kir 2024; Barbera et al. 2025). In a study by Perez et al. (2022), the expression of *EDA2R* was found to be significantly upregulated in the *vastus lateralis* of older adults with frailty compared to healthy older subjects. This study revealed that *EDA2R*, along with other genes like *CDKN1A*, *COL19A1* and *LRRK2*, highlights a distinct subpopulation of senescent myonuclei that appear in aged muscle. The upregulation of *EDA2R* suggests its involvement in chronic muscle dysfunction, contributing to the loss of regenerative capacity and the decline in muscle function commonly associated with ageing.

In a study employing syngeneic tumour models, Bilgic et al. (2023) investigated the role of the *Eda2r* in skeletal muscle wasting driven by cancer cachexia. The researchers found significant upregulation of *Eda2r* mRNA in the skeletal muscles of tumour‐bearing mice compared to controls. Gene expression analyses further revealed elevated *EDA2R* levels in skeletal muscle biopsies from patients with cachectic lung, colorectal, pancreatic and upper gastrointestinal cancers, as well as in muscular dystrophy patients. In vitro experiments demonstrated that treatment of primary mouse and human myotubes with recombinant EDA‐A2 protein induced atrophy, marked by increased expression of atrophy‐related genes (*Atrogin1* and *MuRF1*) and reduced myotube diameter. The study also identified that EDA‐A2 activates non‐canonical NF‐ĸB signalling through NF‐ĸB‐inducing kinase (NIK), promoting muscle protein breakdown. Crucially, *Eda2r*‐knockout mice were resistant to tumour‐induced muscle wasting, preserving muscle mass and function, highlighting the critical role of the EDA‐A2–EDA2R–NIK pathway in cachexia‐associated muscle atrophy. Additionally, the inflammatory cytokine oncostatin M (OSM) upregulated *Eda2r* expression, and muscle‐specific OSM receptor knockout mice were protected from muscle loss, suggesting a synergistic role of OSM–OSMR signalling in this process. These findings position EDA2R‐NIK signalling as a potential therapeutic target for muscle degeneration in cachexia and related atrophic conditions.

In a complementary study, Agca et al. (2024) investigated the role of *Eda2r* in cancer cachexia‐induced muscle atrophy using single‐nucleus RNA sequencing (snRNA‐seq) of tibialis anterior muscles in tumour‐bearing mice. The researchers found that *Eda2r* was significantly upregulated in type IIb myonuclei during cachexia, correlating with increased expression of atrophy‐related genes such as *Atrogin1* and *MuRF1*. Notably, *Eda2r*‐deficient mice were resistant to tumour‐induced muscle wasting and did not exhibit the typical enrichment of type IIb myofibers or the reduction in fibre cross‐sectional area seen in wild‐type mice. Further analysis revealed that *Eda2r* activation in myotubes suppressed oxidative metabolism and muscle contractility pathways, mirroring transcriptional changes observed in cachectic myonuclei. These findings suggest that Eda2r plays a critical role in mediating muscle atrophy by promoting a shift toward type IIb myofiber identity and activating catabolic pathways.

In a recent study by Barbera et al. (2025), the role of EDA2R in age‐associated inflammatory signalling in skeletal muscle was systematically investigated. The authors first demonstrated a strong positive correlation between *EDA2R* mRNA expression and chronological age across skeletal muscle samples from humans, mice and rats. Utilising single‐cell RNA sequencing data from murine and human skeletal muscle, they confirmed that this age‐related upregulation of *EDA2R* occurs across multiple cell types, including myotubes and myogenic precursors such as satellite cells and myoblasts. Next, functional experiments revealed that forced overexpression of *Eda2r* in murine myoblasts led to a marked induction of inflammatory mediators, including *Cxcl5* and *Il6*, closely resembling the transcriptional profile of aged muscle. Similarly, *EDA2R* overexpression in differentiated myotubes resulted in altered expression of genes involved in autophagy, catabolism and muscle development suppression. These effects were conserved in human myoblasts, where *EDA2R* overexpression elicited comparable inflammatory and catabolic responses. Crucially, pharmacological inhibition of EDA2R reversed these changes, significantly attenuating the expression of pro‐inflammatory genes. These findings highlight EDA2R as a key regulator of muscle parainflammatory responses, functionally linking its increased expression to transcriptional changes that mirror ageing‐driven sarcopenia.

Collectively, these studies establish EDA2R as a central mediator of muscle wasting in both sarcopenia and cachexia and highlight its potential as a therapeutic target to mitigate muscle degeneration associated with ageing and chronic illness.

## 7 The Role of EDA2R in Alopecia

### 7.1 Pathophysiology of Alopecia

Alopecia is a condition characterised by the gradual or sudden loss of scalp hair and is often associated with disruptions in the normal hair follicle (HF) cycle, (Jamerson and Aguh 2021; Williams et al. 2021). Hair loss can significantly affect quality of life and poses challenges in both diagnosis and management (Phillips et al. 2017; Stefanato 2010; Goldberg 2023). Ageing‐associated inflammation impairs HF regeneration, and this contributes to progressive hair loss (Liang et al. 2023). Treatment options currently remain limited which highlights the need for improved therapies (Malhotra and Madke 2023; Hordinsky 2021).

**FIGURE 4:**
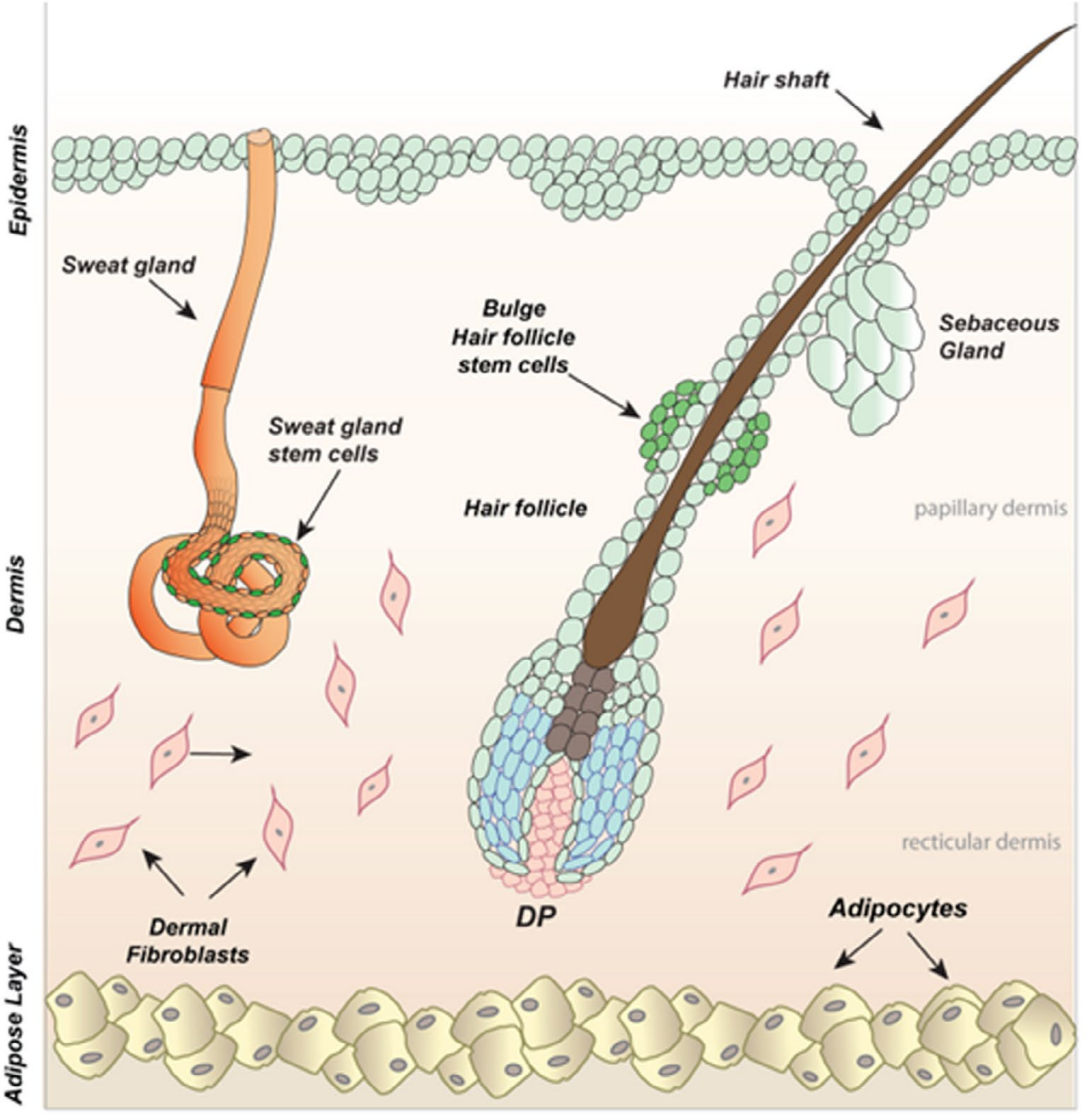
Illustration of a cross‐section of skin with HF. HF resides within the dermis and epidermis of the skin, with adipocytes situated below the HF within the adipose layer (Nicu et al. 2023). Consisting of several components, the HF is a complex structure, often referred to as a ‘miniorgan’ (Cummins et al. 2021). Briefly, the hair shaft, encased by the cuticle, is produced by the HF and composed of compacted dead keratinocytes (Daszczuk et al. 2020). The bulge region houses eHFSCs, which are essential for HF regeneration. Located at the base of the HF, the dermal papilla (DP) is a small cluster of densely packed fibroblasts and is involved in the regulation of factors such as hair shaft length during the growth phase of the hair cycle (Schneider et al. 2009). The sebaceous gland secretes sebum to the epidermal surface which contributes to the waterproofing of hair and skin (Vanderwolf et al. 2023). Image from Daszczuk et al. (2020).

Alopecia is broadly categorised into non‐scarring and scarring types. Non‐scarring forms such as alopecia areata (AA) preserve follicular structure, allowing regrowth (Pratt et al. 2017), whereas scarring alopecias involve irreversible damage to epithelial HF stem cells (eHFSCs) and fibrotic replacement, preventing hair regeneration (Harries and Paus 2010) (Figure 4).

Androgenetic alopecia (AGA) is the most common non‐scarring type and is caused by multiple genes as well as increased sensitivity to androgens, which can lead to follicular miniaturisation (Deng et al. 2023; Xiao et al. 2025; Oiwoh et al. 2024). Mutations in the androgen receptor (*AR*) gene increase sensitivity to dihydrotestosterone (DHT), promoting progressive hair loss (Fu et al. 2021; Wang et al. 2023). A recent GWAS by Sadasivam et al. (2024) emphasised that beyond *AR*, other genes such as *EDA2R* also play an important role in regulating dermal papilla cell function and HF cycling, offering potential therapeutic targets.

### 7.2 EDA2R as a Key Mediator of AGA

The EDA‐A2:EDA2R pathway induces apoptosis and disrupts normal differentiation in hair follicle cells, potentially contributing to follicular regression in AGA (Kwack et al. 2019). Studies in animal models and human cultures have illuminated the roles of EDA2R signalling in hair follicle cycling, highlighting its therapeutic relevance for AGA (Kinde et al. 2024). Prodi et al. (2008) linked *EDA2R* polymorphisms to hair follicle regression via NF‐κB and JNK signalling, while subsequent studies have situated EDA2R within broader networks involving immune responses, hormonal regulation, metabolic dysfunction and systemic ageing (Argentieri et al. 2024; Ntshingila et al. 2023; Lim et al. 2024). These investigations also indicate potential intersections with prostaglandin and Wnt/β‐catenin signalling pathways, further emphasising EDA2R significance in AGA pathogenesis.

Collectively, these findings support EDA2R as a potential biomarker and therapeutic target linking genetic susceptibility, immune modulation and ageing in AGA. As *EDA2R* and the *AR* gene are located in close proximity on the X chromosome, they may be co‐regulated or co‐expressed in follicular cells (Redler et al. 2012). Current treatments targeting AR, such as finasteride and spironolactone, have unclear effects on EDA2R expression (Zhou et al. 2022). Given the role of EDA2R in pathways such as NF‐κB and follicular cycling (Benitez et al. 2024), further investigation may reveal additional therapeutic opportunities in AGA.

### 7.3 Current and Emerging Therapies for AGA: Targeting EDA2R

Currently, topical minoxidil and oral finasteride are the only FDA‐approved treatments for AGA (Devjani et al. 2023). Minoxidil enhances blood flow to hair follicles, thereby prolonging the anagen phase, while finasteride reduces DHT levels, slowing hair loss and promoting regrowth (Hussein et al. 2024; Piraccini et al. 2022). Dutasteride, although not licenced for AGA, acts similarly by reducing DHT through inhibition of 5 alpha reductase (Dominguez‐Santas et al. 2022). Other androgen receptor antagonists such as flutamide, bicalutamide and spironolactone have demonstrated efficacy but are limited by their off‐label status and potential adverse effects (Varothai and Bergfeld 2014; Abdel‐Raouf et al. 2021; Perez et al. 2025; Desai et al. 2024). Emerging approaches, including clascoterone and nanocarrier‐based therapies, are under investigation to enhance drug delivery and safety (Eichenfield et al. 2020; Pozo‐Pérez et al. 2024).

Wnt/β‐catenin signalling and prostaglandin D2 have emerged as key pathways in AGA pathogenesis (Ryu et al. 2022; Garza et al. 2012). Therapies under investigation include cetirizine, stem cell exosomes, platelet‐rich plasma and gene‐targeted approaches to restore follicular function (Abdin et al. 2022; Papukashvili et al. 2021; Tang et al. 2023; Zaky et al. 2021). Natural compounds such as caffeine and saw palmetto show potential antiandrogenic or follicle‐stimulating effects, though further validation is needed (Elnady et al. 2025; Evron et al. 2020). In this context, it is essential to determine whether existing or future pharmacological agents can either downregulate *EDA2R* gene expression or directly inhibit EDA2R receptor activity at the protein level, both of which represent promising strategies for counteracting hair follicle regression and promoting regeneration in AGA.

## 8 Modifiable Factors and Therapeutic Strategies Targeting EDA2R

While direct EDA2R antagonists are not yet available, multiple approaches have been identified to achieve this suppression indirectly. Among these, nutritional interventions, particularly those involving caloric restriction, have gained increasing attention as feasible modulators of EDA2R levels. Pietzner et al. (2024) found that 7‐day water‐only fasting triggered a multi‐organ proteomic response, including a significant reduction in plasma EDA2R protein levels (*p* = 0.0013). This is in line with the study by Lund et al. (2024), who employed targeted plasma proteomics to identify circulating factors of overfeeding in mice and found that EDA2R was among six proteins significantly affected, with its levels upregulated in response to overfeeding. Taken together, these findings suggest that EDA2R protein levels are sensitive to nutritional status and may play a key role in mediating metabolic and inflammatory responses to both fasting and overfeeding.

Separately, Lee et al. (2025) demonstrated that Ginkgolide B (GB) treatment in aged female mice reversed the expression of ageing‐associated genes, including *Eda2r* (*p* < 0.05), suggesting GB's potential to mitigate age‐related inflammation. The downregulation of *Eda2r* by GB may prevent muscle atrophy, and reduce systemic stress responses. Importantly, this represents the first interventional study to show that *Eda2r* can be pharmacologically downregulated by a naturally derived molecule.

Further supporting the role of metabolic modulators, Geng et al. (2025) identified betaine as a key exercise‐induced metabolite with strong geroprotective effects. In aged mouse muscle, expression of the *Eda2r* gene was markedly upregulated compared to that in young mice; however, this increase was effectively reversed by oral betaine administration. Supplementation of aged mice with 0.1%–1% (w/v) betaine in drinking water for 3 weeks–3 months significantly reduced *Eda2r* expression (adjusted *p* = 0.009) relative to aged controls. Collectively, these findings indicate that betaine, an endogenous metabolite elevated by long‐term exercise, can systemically counteract age‐associated inflammatory gene expression at the molecular level.

Conversely, the study by Fernandez‐Gonzalo et al. (2020) demonstrated that prolonged physical inactivity resulting from 84 days of bed rest in healthy men led to a significant (*p* = 0.031) upregulation of *EDA2R* gene expression in human skeletal muscle (vastus lateralis), suggesting a link between disuse‐induced muscle atrophy and increased *EDA2R* expression. In agreement with this, *Eda2r* expression in skeletal muscle was found to be significantly (*p* = 0.02) lower in exercised compared to sedentary mice (Moore et al. 2023), indicating that physical activity may suppress *Eda2r* expression. Furthermore, the study by Taylor et al. (2025), which included 20 healthy untrained males and 20 healthy untrained females, found that *EDA2R* gene expression was significantly downregulated at 3 (adjusted *p* = 0.005), 6 (adjusted *p* = 0.025) and 9 (adjusted *p* = 0.042) hours after high‐intensity interval exercise. Together, these observations support the notion that *EDA2R* contributes to the molecular response to muscle disuse, and its downregulation may represent one of the mechanisms through which exercise protects against muscle atrophy and related degenerative processes.

Finally, the study by Margara‐Escudero et al. (2025) analysed 314 older adults with overweight/obesity and metabolic syndrome enrolled in the 6‐year PREDIMED‐Plus trial, using plasma neurology‐related proteins, alongside repeated cognitive assessments. They identified protein signatures predictive of cognitive decline, highlighting seven proteins, including EDA2R, consistently associated across multiple cognitive domains. Lifestyle interventions involving weight loss, a Mediterranean diet and physical activity modulated these associations, suggesting that EDA2R may serve as a modifiable biomarker linking lifestyle to cognitive health.

**FIGURE 5:**
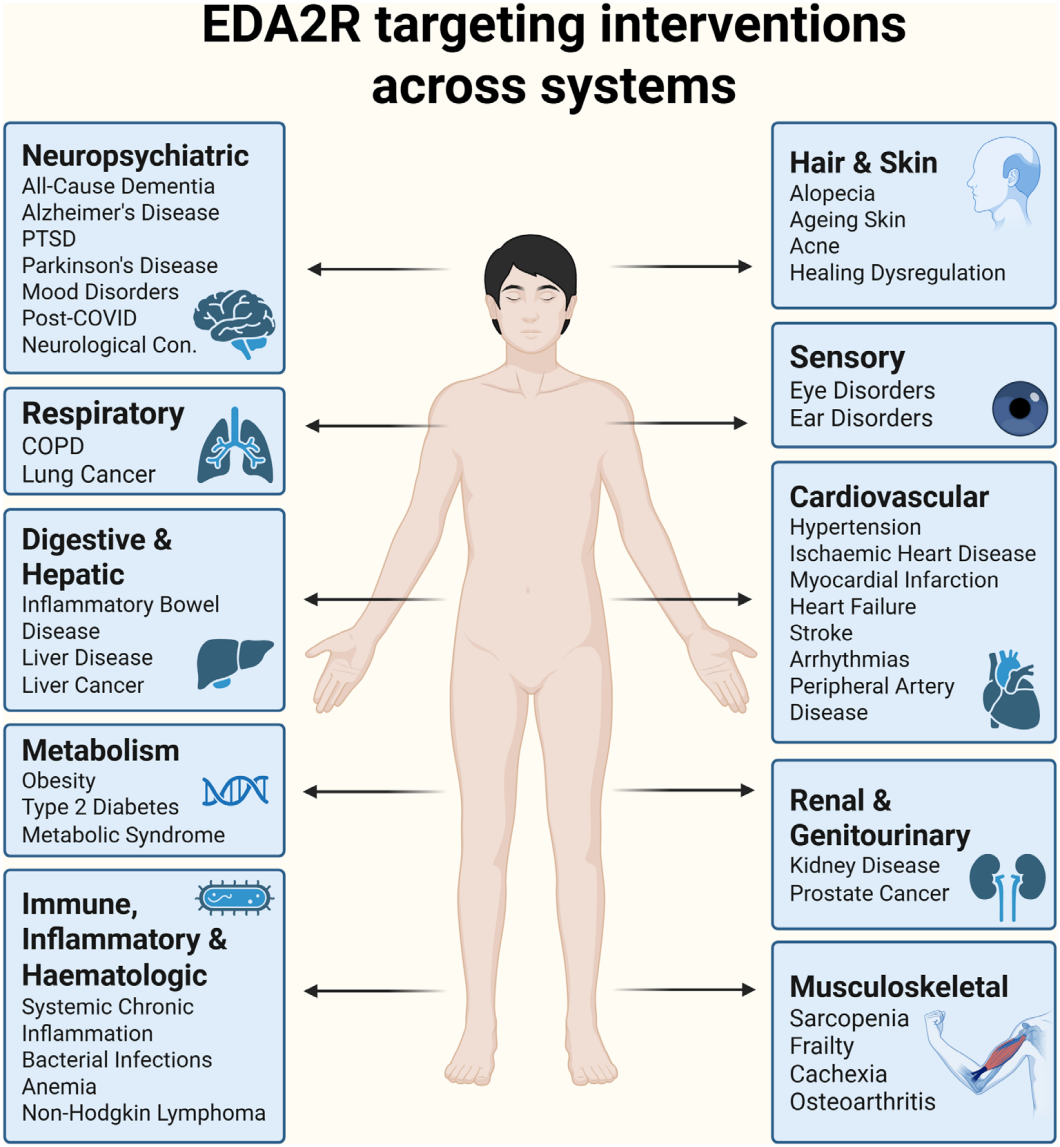
Illustrations of EDA2R‐targeting interventions across systems. This figure illustrates potential therapeutic intervention points for targeting EDA2R across multiple human body systems. The systems and associated diseases depicted here are derived from our current review of experimental and clinical studies on EDA2R. EDA2R plays a key role in inflammation, apoptosis, NF‐κB/JNK signalling and tissue remodelling, making it a relevant target across diverse physiological and pathological contexts. Anatomical icons are paired with system‐specific callout boxes that detail conditions and functions potentially influenced by EDA2R modulation. Systems represented include: The central nervous system (brain), linked to neuroinflammation and neurodegenerative disorders; hair follicles, implicated in ectodermal dysplasia; the cardiovascular system, highlighting vascular inflammation and remodelling; skeletal muscle, noting atrophy and regeneration; metabolic tissues, including adipose tissue, associated with metabolic dysregulation; and the integumentary system (skin), associated with barrier dysfunction and inflammatory skin conditions. The figure provides a conceptual framework supporting the rationale for system‐specific EDA2R‐based therapeutic strategies. (Illustration made in BioRender).

In summary, these studies highlight the potential of interventions such as fasting, Ginkgolide B, betaine and exercise to modulate EDA2R, emphasising its emerging role in inflammation, muscle health and ageing. Figure 5 illustrates potential therapeutic intervention points across multiple human body systems. Future research should further investigate these and other strategies in animal models and human clinical settings, as well as assess how established geroprotective compounds influence EDA2R regulation. Developing and evaluating targeted therapies, including receptor antagonists or RNA‐based approaches to suppress EDA2R expression, will be critical to harness its full therapeutic potential.

## 9 Challenges, Future Perspectives and Conclusions

Current understanding of EDA2R biology and its clinical relevance is limited by structural and mechanistic gaps. Despite growing evidence for its role as an ageing biomarker, the molecular structure and ligand interactions of EDA2R remain poorly defined. A key limitation is the absence of an experimentally resolved crystal structure for the EDA‐A2:EDA2R complex, leaving key ligand‐binding interfaces and receptor activation mechanisms unknown. Although the extracellular domains of EDA2R and EDA‐A2 have been individually characterised, their interaction dynamics are unclear, especially compared to the well‐studied EDAR:EDA‐A1 complex. Current insights rely largely on computational predictions, such as AlphaFold3 models, which, while informative, lack experimental validation. Downstream signalling is also poorly understood; unlike EDAR, which recruits EDARADD to activate NF‐κB, EDA2R appears not to involve EDARADD, and potential alternative adaptor proteins remain unidentified. The absence of selective EDA2R antagonists further limits mechanistic and pharmacological studies. Future structural and functional research is needed to elucidate receptor–ligand specificity and enable targeted modulation of EDA2R signalling.

Research on EDA2R often relies on preclinical models and associative data, reducing translational potential. In muscle ageing and cachexia, evidence comes mainly from animal and in vitro studies, with Barbera et al. (2025) providing multi‐tissue human transcriptomics and myotube data implicating EDA2R in parainflammatory ageing. Lack of in vivo human validation, accelerated ageing models and longitudinal data constrain clinical translation. While EDA2R is critical for embryonic ectodermal development (Mikkola 2008; Lee and Tumbar 2012) and may influence sperm capacitation (Anjorin et al. 2025), the optimal window for safe targeting in age‐related pathologies is unknown, as premature suppression could disrupt physiological roles.

Clinical research is largely observational, examining associations between EDA2R expression or protein levels and various conditions. A key limitation in this area is the underrepresentation of non‐European populations in genetic and clinical studies, which restricts the generalisability of current findings. The scarcity of mechanistic studies, longitudinal cohorts and interventional trials limits insight into its predictive and therapeutic potential. In dermatology, studies are mostly in vitro or in animals, and conditions such as acne or androgenetic alopecia are further complicated by limited clinical validation, homogeneous populations and insufficient functional characterisation within broader signalling networks.

An additional limitation of the present review is its predominantly positive narrative regarding EDA2R. The available evidence was gathered mainly through searches using EDA2R as a keyword, which may introduce selection bias by favouring studies reporting significant findings. Negative or null results may remain unpublished or confined to supplementary datasets. Future systematic reviews and meta‐analyses should therefore aim to interrogate full datasets, including supplementary materials, to provide a more balanced evaluation. Another limitation is that X‐chromosomal genes like *EDA2R* are often excluded from genome‐wide association studies and other large‐scale analyses, resulting in underrepresentation in current genetic literature. In addition, because EDA2R expression depends on age, future studies should adjust analyses for age to avoid confounding.

Furthermore, indirect modulation strategies targeting EDA2R, including physical activity, caloric restriction, betaine and Ginkgolide B, suffer from limited mechanistic focus, lack of clinical trials and absence of long‐term outcome data, further constrained by sex‐ and species‐specific effects. Collectively, these limitations highlight the urgent need for high‐resolution structural studies, comprehensive longitudinal human data, mechanistic validation in clinically relevant tissues and rigorously designed clinical trials. Addressing these gaps will be essential to elucidate the precise biological functions of EDA2R and realise its therapeutic potential across multiple disease domains.

Although this review highlights EDA2R as a promising biomarker of ageing and disease, it is important to recognise that it is not unique in this regard. Transcriptomic and proteomic studies that identified EDA2R among the top ageing‐associated molecules also revealed other strong candidates, such as growth differentiation factor 15 (GDF15), which consistently associates with chronological and biological ageing across multiple tissues (Barbera et al. 2025; Arif et al. 2025; Tian et al. 2025). GDF15, a stress‐responsive cytokine of the Transforming Growth Factor beta (TGF‐β) superfamily, has emerged as a robust biomarker and potential therapeutic target in metabolic, cardiovascular and age‐related diseases (Tian et al. 2025). Other well‐established ageing biomarkers identified in multiple studies include A2M (Alpha‐2‐macroglobulin), ADIPO (Adiponectin), AHSG (Alpha‐2‐HS‐Glycoprotein), B2M (Beta‐2‐Microglobulin), CLU (Clusterin), CRP (C‐reactive protein), CST3 (Cystatin C), CTSB (Cathepsin B), GHR (Growth Hormone Receptor), IGF‐1 (Insulin‐like Growth Factor 1), IGFBP2 (Insulin‐like Growth Factor Binding Protein 2), IL‐6 (Interleukin‐6), Klotho (Klotho), NPPB (Natriuretic Peptide B), PTN (Pleiotrophin), SERPINA3 (Alpha‐1‐Antichymotrypsin), SIRT1 (Sirtuin 1), SIRT3 (Sirtuin 3), SHBG (Sex Hormone‐Binding Globulin), TIMP1 (Metalloproteinase Inhibitor 1), TNF‐α (Tumour Necrosis Factor alpha), TNFR1 (Tumour Necrosis Factor Receptor 1) and VEGFA (Vascular Endothelial Growth Factor A). These findings suggest that, while EDA2R shows strong potential as an integrative biomarker of ageing, its specificity and predictive power should be assessed alongside other established markers, including GDF15, to fully determine its clinical utility and translational relevance.

In conclusion, EDA2R plays a central role in ageing and inflammation, acting as a mediator of inflammaging. Its expression increases with age across multiple tissues, promoting chronic parainflammatory states via canonical and non‐canonical NF‐κB signalling. Elevated levels are linked to systemic ageing, cellular senescence and diverse age‐related conditions. Genetic and proteomic data highlight tissue‐specific roles in muscle degeneration and ectodermal maintenance, indicating its function as both a biomarker and driver of ageing. Although pharmacological antagonists are lacking, lifestyle interventions such as caloric restriction and exercise may modulate EDA2R, making it a promising target to extend healthspan and mitigate age‐related diseases.

## Author Contributions

I.I.A. and K.R. conceived the concept of the review. G.F., L.T., T.B., S.S., C.F., L.L., S.K., M.B., I.I.A. and K.R. conducted the literature search and analysis. G.F. and I.I.A. drafted the original manuscript. G.F. prepared Figures 3 and 5. L.T., T.B., S.S., C.F., L.L., S.K., M.B., I.I.A. and K.R. contributed to revising and editing the manuscript. All authors read and approved the final version of the manuscript.

## Conflicts of Interest

The authors declare no conflicts of interest.

## Data Availability

Data sharing not applicable to this article as no datasets were generated or analysed during the current study.

## References

[acel70282-bib-0001] Abdel‐Raouf, H. , U. F. Aly , W. Medhat , S. S. Ahmed , and R. T. A. Abdel‐Aziz . 2021. “A Novel Topical Combination of Minoxidil and Spironolactone for Androgenetic Alopecia: Clinical, Histopathological, and Physicochemical Study.” Dermatologic Therapy 34, no. 1: e14678. 10.1111/dth.14678. 33320406

[acel70282-bib-0195] Abdin, R. , Y. Zhang , and J. J. Jimenez . 2022. “Treatment of Androgenetic Alopecia Using PRP to Target Dysregulated Mechanisms and Pathways.” Front Med (Lausanne) 9: 843127. 10.3389/fmed.2022.843127. 35372424 PMC8965895

[acel70282-bib-0002] Abramson, J. , J. Adler , J. Dunger , et al. 2024. “Accurate Structure Prediction of Biomolecular Interactions With AlphaFold 3.” Nature 630, no. 8016: 493–500. 10.1038/s41586-024-07487-w. 38718835 PMC11168924

[acel70282-bib-0003] Afzal, S. , A. S. Abdul Manap , A. Attiq , I. Albokhadaim , M. Kandeel , and S. M. Alhojaily . 2023. “From Imbalance to Impairment: The Central Role of Reactive Oxygen Species in Oxidative Stress‐Induced Disorders and Therapeutic Exploration.” Frontiers in Pharmacology 14: 1269581. 37927596 10.3389/fphar.2023.1269581 PMC10622810

[acel70282-bib-0004] Agca, S. , A. Domaniku‐Waraich , S. N. Bilgic , et al. 2024. “Tumour‐Induced Alterations in Single‐Nucleus Transcriptome of Atrophying Muscles Indicate Enhanced Protein Degradation and Reduced Oxidative Metabolism.” Journal of Cachexia, Sarcopenia and Muscle 15, no. 5: 1898–1914. 39001644 10.1002/jcsm.13540 PMC11446705

[acel70282-bib-0005] Anjorin, O. I. , T. Yamanaka , and M. Shimada . 2025. “Functions of Ectodysplasin A2 Receptor (EDA2R) in Inducing Capacitation of Sperm in Mice.” In Vitro Cellular & Developmental Biology. Animal. 10.1007/s11626-025-01084-5. PMC12589252 40691399

[acel70282-bib-0006] Argentieri, M. A. , S. Xiao , D. Bennett , et al. 2024. “Proteomic Aging Clock Predicts Mortality and Risk of Common Age‐Related Diseases in Diverse Populations.” Nature Medicine 30, no. 9: 2450–2460. 10.1038/s41591-024-03164-7 PMC11405266 39117878

[acel70282-bib-0007] Arif, M. , A. Lehoczki , G. Haskó , F. W. Lohoff , Z. Ungvari , and P. Pacher . 2025. “Global and Tissue‐Specific Transcriptomic Dysregulation in Human Aging: Pathways and Predictive Biomarkers.” GeroScience 47: 1–20. 40295347 10.1007/s11357-025-01672-z PMC12397481

[acel70282-bib-0008] Aryankalayil, M. J. , M. A. Bylicky , S. Martello , et al. 2023. “Microarray Analysis Identifies Coding and Non‐Coding RNA Markers of Liver Injury in Whole Body Irradiated Mice.” Scientific Reports 13, no. 1: 200. 10.1038/s41598-022-26784-w. 36604457 PMC9814510

[acel70282-bib-0009] Baechle, J. J. , N. Chen , P. Makhijani , S. Winer , D. Furman , and D. A. Winer . 2023. “Chronic Inflammation and the Hallmarks of Aging.” Molecular Metabolism 74: 101755. 37329949 10.1016/j.molmet.2023.101755 PMC10359950

[acel70282-bib-0010] Baek, M. , F. DiMaio , I. Anishchenko , et al. 2021. “Accurate Prediction of Protein Structures and Interactions Using a Three‐Track Neural Network.” Science 373, no. 6557: 871–876. 10.1126/science.abj8754. 34282049 PMC7612213

[acel70282-bib-0011] Barbera, M. C. , L. Guarrera , A. D. Re Cecconi , et al. 2025. “Increased Ectodysplasin‐A2‐Receptor EDA2R Is a Ubiquitous Hallmark of Aging and Mediates Parainflammatory Responses.” Nature Communications 16, no. 1: 1898. 10.1038/s41467-025-56918-3. PMC11847917 39988718

[acel70282-bib-0012] Barnabei, L. , E. Laplantine , W. Mbongo , F. Rieux‐Laucat , and R. Weil . 2021. “NF‐κB: At the Borders of Autoimmunity and Inflammation.” Frontiers in Immunology 12: 716469. 34434197 10.3389/fimmu.2021.716469 PMC8381650

[acel70282-bib-0013] Baumert, P. , S. Mäntyselkä , M. Schönfelder , et al. 2024. “Skeletal Muscle Hypertrophy Rewires Glucose Metabolism: An Experimental Investigation and Systematic Review.” Journal of Cachexia, Sarcopenia and Muscle 15, no. 3: 989–1002. 10.1002/jcsm.13468. 38742477 PMC11154753

[acel70282-bib-0014] Benitez, M. B. M. , Y. P. Navarro , E. Azuara‐Liceaga , A. T. Cruz , J. V. Flores , and L. Lopez‐Canovas . 2024. “Circular RNAs and the Regulation of Gene Expression in Diabetic Nephropathy.” International Journal of Molecular Medicine 53, no. 5: 44. 38516776 10.3892/ijmm.2024.5368 PMC10998718

[acel70282-bib-0015] Bergqvist, C. , P. Ramia , O. Abbas , and M. Kurban . 2017. “Genetics of Syndromic and Non‐Syndromic Hereditary Nail Disorders.” Clinical Genetics 91, no. 6: 813–823. 27613389 10.1111/cge.12852

[acel70282-bib-0016] Bilgic, S. N. , A. Domaniku , B. Toledo , et al. 2023. “EDA2R–NIK Signalling Promotes Muscle Atrophy Linked to Cancer Cachexia.” Nature 617, no. 7962: 827–834. 37165186 10.1038/s41586-023-06047-y

[acel70282-bib-0017] Biomarkers of Aging Consortium , Herzog, C. M. S. , L. J. E. Goeminne , J. R. Poganik , et al. 2024. “Challenges and Recommendations for the Translation of Biomarkers of Aging.” Nature Aging 4, no. 10: 1372–1383. 10.1038/s43587-024-00683-3. 39285015 PMC12262637

[acel70282-bib-0019] Bueno Álvez, M. , S. Bergström , J. Kenrick , et al. 2025. “A Human Pan‐Disease Blood Atlas of the Circulating Proteome.” Science: eadx2678. 10.1126/science.adx2678. 41066540

[acel70282-bib-0020] Burley, S. K. , H. M. Berman , J. M. Duarte , et al. 2022. “Protein Data Bank: A Comprehensive Review of 3D Structure Holdings and Worldwide Utilization by Researchers, Educators, and Students.” Biomolecules 12, no. 10: 1425. 10.3390/biom12101425. 36291635 PMC9599165

[acel70282-bib-0021] Burley, S. K. , H. M. Berman , G. J. Kleywegt , J. L. Markley , H. Nakamura , and S. Velankar . 2017. “Protein Data Bank (PDB): The Single Global Macromolecular Structure Archive.” Methods in Molecular Biology 1607: 627–641. 10.1007/978-1-4939-7000-1_26. 28573592 PMC5823500

[acel70282-bib-0022] Cai, Z. , X. Deng , J. Jia , D. Wang , and G. Yuan . 2021. “Ectodysplasin A/Ectodysplasin A Receptor System and Their Roles in Multiple Diseases.” Frontiers in Physiology 12: 788411. 34938205 10.3389/fphys.2021.788411 PMC8685516

[acel70282-bib-0023] Calder, P. C. , N. Bosco , R. Bourdet‐Sicard , et al. 2017. “Health Relevance of the Modification of Low Grade Inflammation in Ageing (Inflammageing) and the Role of Nutrition.” Ageing Research Reviews 40: 95–119. 28899766 10.1016/j.arr.2017.09.001

[acel70282-bib-0024] Ceruti, J. M. , G. J. Leirós , and M. E. Balañá . 2018. “Androgens and Androgen Receptor Action in Skin and Hair Follicles.” Molecular and Cellular Endocrinology 465: 122–133. 28912032 10.1016/j.mce.2017.09.009

[acel70282-bib-0025] Chen, Y. L. , J. J. Wang , J. You , et al. 2025. “Systematic Analyses Uncover Plasma Proteins Linked to Incident Cardiovascular Diseases.” Protein & Cell 2025: pwaf072. 10.1093/procel/pwaf072. PMC12987571 40927895

[acel70282-bib-0026] Cummins, D. M. , I. H. Chaudhry , and M. Harries . 2021. “Scarring Alopecias: Pathology and an Update on Digital Developments.” Biomedicine 9: 1755. 10.3390/biomedicines9121755 PMC8698437 34944572

[acel70282-bib-0027] Daszczuk, P. , P. Mazurek , T. D. Pieczonka , A. Olczak , L. M. Boryń , and K. Kobielak . 2020. “An Intrinsic Oscillation of Gene Networks Inside Hair Follicle Stem Cells: An Additional Layer That Can Modulate Hair Stem Cell Activities.” Frontiers in Cell and Developmental Biology 8: 595178. 33363148 10.3389/fcell.2020.595178 PMC7758224

[acel70282-bib-0028] Daubert, M. A. , and A. Jeremias . 2010. “The Utility of Troponin Measurement to Detect Myocardial Infarction: Review of the Current Findings.” Vascular Health and Risk Management 6: 691–699. 10.2147/vhrm.s5306. 20859540 PMC2941782

[acel70282-bib-0029] Deng, Y. , M. Wang , Y. He , F. Liu , L. Chen , and X. Xiong . 2023. “Cellular Senescence: Ageing and Androgenetic Alopecia.” Dermatology 239, no. 4: 533–541. 37088073 10.1159/000530681

[acel70282-bib-0030] Desai, D. D. , A. Nohria , M. Sikora , N. Anyanwu , J. Shapiro , and K. I. Lo Sicco . 2024. “Comparative Analysis of Low‐Dose Oral Minoxidil With Spironolactone Versus Finasteride or Dutasteride in Female Androgenetic Alopecia Management.” Archives of Dermatological Research 316, no. 9: 622. 10.1007/s00403-024-03361-x. 39276230

[acel70282-bib-0031] Deshmukh, S. , and S. Prashanth . 2012. “Ectodermal Dysplasia: A Genetic Review.” International Journal of Clinical Pediatric Dentistry 5, no. 3: 197–202. 25206167 10.5005/jp-journals-10005-1165 PMC4155886

[acel70282-bib-0032] Devjani, S. , O. Ezemma , K. J. Kelley , E. Stratton , and M. Senna . 2023. “Androgenetic Alopecia: Therapy Update.” Drugs 83, no. 8: 701–715. 37166619 10.1007/s40265-023-01880-x PMC10173235

[acel70282-bib-0196] Dominguez‐Santas, M. , B. Diaz‐Guimaraens , D. Saceda‐Corralo , et al. 2022. “The State‐of‐the‐art in the Management of Androgenetic Alopecia: A Review of New Therapies and Treatment Algorithms.” Jeadv Clinical Practice 1, no. 3: 176–185. 10.1002/jvc2.53.

[acel70282-bib-0034] Dorgaleleh, S. , K. Naghipoor , Z. Hajimohammadi , and M. Oladnab . 2021. “Molecular Basis of Ectodermal Dysplasia: A Comprehensive Review of the Literature.” Egyptian Journal of Dermatology and Venereology 41, no. 2: 55–66.

[acel70282-bib-0035] Dostert, C. , M. Grusdat , E. Letellier , and D. Brenner . 2019. “The TNF Family of Ligands and Receptors: Communication Modules in the Immune System and Beyond.” Physiological Reviews 99, no. 1: 115–160. 30354964 10.1152/physrev.00045.2017

[acel70282-bib-0036] Eichenfield, L. , A. Hebert , L. S. Gold , et al. 2020. “Open‐Label, Long‐Term Extension Study to Evaluate the Safety of Clascoterone (CB‐03‐01) Cream, 1% Twice Daily, in Patients With Acne Vulgaris.” Journal of the American Academy of Dermatology 83, no. 2: 477–485. 32348828 10.1016/j.jaad.2020.04.087

[acel70282-bib-0037] Elnady, R. E. , M. S. Abdon , H. R. Shaheen , R. M. Eladawy , Y. O. Azar , and S. M. Al Raish . 2025. “The Future of Alopecia Treatment: Plant Extracts, Nanocarriers, and 3D Bioprinting in Focus.” Pharmaceutics 17, no. 5: 584. 40430875 10.3390/pharmaceutics17050584 PMC12115063

[acel70282-bib-0038] Elsworth, J. D. , A. Neutzner , J. Roux , et al. 2025. “Proteomic Signatures of Epigenetic Age in African Green Monkey Cerebrospinal Fluid and Plasma.” Aging Cell 24: e70168. 10.1111/acel.70168. 40729555 PMC12507418

[acel70282-bib-0039] Ensembl . 2025. “The Ensembl Project Portal.” Accessed on 17 June 2025. http://www.ensembl.org/Homo_sapiens/Info/Index.

[acel70282-bib-0040] Evron, E. , M. Juhasz , A. Babadjouni , and N. A. Mesinkovska . 2020. “Natural Hair Supplement: Friend or Foe? Saw Palmetto, a Systematic Review in Alopecia.” Skin Appendage Disorders 6, no. 6: 329–337. 10.1159/000509905. 33313047 PMC7706486

[acel70282-bib-0041] Feng, L. T. , Z. N. Chen , and H. Bian . 2024. “Skeletal Muscle: Molecular Structure, Myogenesis, Biological Functions, and Diseases.” MedComm 5, no. 7: e649. 38988494 10.1002/mco2.649 PMC11234433

[acel70282-bib-0042] Fernandez‐Gonzalo, R. , P. A. Tesch , T. R. Lundberg , B. A. Alkner , E. Rullman , and T. Gustafsson . 2020. “Three Months of Bed Rest Induce a Residual Transcriptomic Signature Resilient to Resistance Exercise Countermeasures.” FASEB Journal 34, no. 6: 7958–7969. 10.1096/fj.201902976R. 32293758

[acel70282-bib-0043] Ferreira, L. G. , R. N. Dos Santos , G. Oliva , and A. D. Andricopulo . 2015. “Molecular Docking and Structure‐Based Drug Design Strategies.” Molecules 20, no. 7: 13384–13421. 26205061 10.3390/molecules200713384 PMC6332083

[acel70282-bib-0044] Fu, D. , J. Huang , K. Li , et al. 2021. “Dihydrotestosterone‐Induced Hair Regrowth Inhibition by Activating Androgen Receptor in C57BL6 Mice Simulates Androgenetic Alopecia.” Biomedicine & Pharmacotherapy 137: 111247. 33517191 10.1016/j.biopha.2021.111247

[acel70282-bib-0045] Gao, Y. , X. Jiang , Z. Wei , H. Long , and W. Lai . 2023. “The EDA/EDAR/NF‐κB Pathway in Non‐Syndromic Tooth Agenesis: A Genetic Perspective.” Frontiers in Genetics 14: 1168538. 37077539 10.3389/fgene.2023.1168538 PMC10106650

[acel70282-bib-0197] Garza, L. A. , Y. Liu , Z. Yang , et al. 2012. “Prostaglandin D2 Inhibits Hair Growth and is Elevated in Bald Scalp of Men with Androgenetic Alopecia.” Science Translational Medicine 4, no. 126: 126ra134. 10.1126/scitranslmed.3003122. PMC3319975 22440736

[acel70282-bib-0046] Geng, L. , J. Ping , R. Wu , et al. 2025. “Systematic Profiling Reveals Betaine as an Exercise Mimetic for Geroprotection.” Cell 188, no. 19: 5426–5428. 10.1016/j.cell.2025.07.030. 40803320

[acel70282-bib-0047] Giavarina, D. , F. Husain‐Syed , and C. Ronco . 2021. “Clinical Implications of the New Equation to Estimate Glomerular Filtration Rate.” Nephron 145, no. 5: 508–512. 10.1159/000516638. 34120119

[acel70282-bib-0048] Ginevičienė, V. , E. Pranckevičienė , A. Urnikytė , et al. 2025. “A Multi‐Omics Investigation of Sarcopenia and Frailty: Integrating Genomic, Epigenomic and Telomere Length Data.” Experimental Physiology: 1–17. 10.1113/EP092853. 41015547 PMC13394718

[acel70282-bib-0049] Glass, D. J. 2003. “Signalling Pathways That Mediate Skeletal Muscle Hypertrophy and Atrophy.” Nature Cell Biology 5, no. 2: 87–90. 10.1038/ncb0203-87. 12563267

[acel70282-bib-0050] Goldberg, L. J. 2023. “Alopecia–New Building Blocks.” Journal of the American Academy of Dermatology 89, no. 2: S1–S2. 37591559 10.1016/j.jaad.2023.05.048

[acel70282-bib-0051] Gong, J. , D. M. Williams , S. Scholes , et al. 2025. “Unraveling the Role of Proteins in Dementia: Insights From Two UK Cohorts With Causal Evidence.” Brain Communications 7, no. 2: fcaf097. 40092369 10.1093/braincomms/fcaf097 PMC11906402

[acel70282-bib-0052] Google DeepMind, & EMBL‐EBI . 2025. AlphaFold Protein Structure Database. Google.

[acel70282-bib-0054] GTEx Portal . Accessed on 17 June 2025. https://gtexportal.org/home/index.html. 2025.

[acel70282-bib-0055] Guan, Z. H. , D. Yang , Y. Wang , J. B. Ma , and G. N. Wang . 2024. “Ectodysplasin‐A2 Receptor (EDA2R) Knockdown Alleviates Myocardial Ischemia/Reperfusion Injury Through Inhibiting the Activation of the NF‐κB Signalling Pathway.” Experimental Animals 73, no. 4: 376–389. 38797667 10.1538/expanim.24-0020 PMC11534487

[acel70282-bib-0057] Guo, J. , X. Huang , L. Dou , et al. 2022. “Aging and Aging‐Related Diseases: From Molecular Mechanisms to Interventions and Treatments.” Signal Transduction and Targeted Therapy 7, no. 1: 391. 36522308 10.1038/s41392-022-01251-0 PMC9755275

[acel70282-bib-0058] Guo, Y. , J. You , Y. Zhang , et al. 2024. “Plasma Proteomic Profiles Predict Future Dementia in Healthy Adults.” Nature Aging 4, no. 2: 247–260. 10.1038/s43587-023-00565-0. 38347190

[acel70282-bib-0059] Hajam, Y. A. , R. Rani , S. Y. Ganie , et al. 2022. “Oxidative Stress in Human Pathology and Aging: Molecular Mechanisms and Perspectives.” Cells 11, no. 3: 552. 35159361 10.3390/cells11030552 PMC8833991

[acel70282-bib-0060] Halper‐Stromberg, A. , and B. Jabri . 2022. “Maladaptive Consequences of Inflammatory Events Shape Individual Immune Identity.” Nature Immunology 23, no. 12: 1675–1686. 36411382 10.1038/s41590-022-01342-8

[acel70282-bib-0061] Harries, M. J. , and R. Paus . 2010. “The Pathogenesis of Primary Cicatricial Alopecias.” American Journal of Pathology 177: 2152–2162. 20889564 10.2353/ajpath.2010.100454 PMC2966773

[acel70282-bib-0062] Harris, S. E. , S. R. Cox , S. Bell , et al. 2020. “Neurology‐Related Protein Biomarkers Are Associated With Cognitive Ability and Brain Volume in Older Age.” Nature Communications 11, no. 1: 800. 10.1038/s41467-019-14161-7 PMC7010796 32041957

[acel70282-bib-0063] Henne, S. K. , R. Aldisi , S. Sivalingam , et al. 2023. “Analysis of 72,469 UK Biobank Exomes Links Rare Variants to Male‐Pattern Hair Loss.” Nature Communications 14, no. 1: 5492. 10.1038/s41467-023-41186-w. PMC10517150 37737258

[acel70282-bib-0064] Heo, L. , G. Janson , and M. Feig . 2021. “Physics‐Based Protein Structure Refinement in the Era of Artificial Intelligence.” Proteins: Structure, Function, and Bioinformatics 89, no. 12: 1870–1887. 10.1002/prot.26161 PMC8616793 34156124

[acel70282-bib-0065] Hordinsky, M. 2021. “Scarring Alopecia: Diagnosis and New Treatment Options.” Dermatologic Clinics 39: 383–388. 34053592 10.1016/j.det.2021.05.001

[acel70282-bib-0066] Huang, P.‐S. , K. Feldmeier , F. Parmeggiani , D. A. Fernandez Velasco , B. Höcker , and D. Baker . 2016. “De Novo Design of a Four‐Fold Symmetric TIM‐Barrel Protein With Atomic‐Level Accuracy.” Nature Chemical Biology 12, no. 1: 29–34. 26595462 10.1038/nchembio.1966 PMC4684731

[acel70282-bib-0067] Hussein, R. S. , S. B. Dayel , O. Abahussein , and A. A. El‐Sherbiny . 2024. “Applications and Efficacy of Minoxidil in Dermatology.” Skin Health and Disease 4, no. 6: e472. 39624749 10.1002/ski2.472 PMC11608877

[acel70282-bib-0068] Hymowitz, S. G. , D. M. Compaan , M. Yan , et al. 2003. “The Crystal Structures of EDA‐A1 and EDA‐A2: Splice Variants With Distinct Receptor Specificity.” Structure 11, no. 12: 1513–1520. 14656435 10.1016/j.str.2003.11.009

[acel70282-bib-0203] Iakovou, E. , and M. Kourti . 2022. “A Comprehensive Overview of the Complex Role of Oxidative Stress in Aging, the Contributing Environmental Stressors and Emerging Antioxidant Therapeutic Interventions.” Frontiers in Aging Neuroscience 14: 827900. 10.3389/fnagi.2022.827900. 35769600 PMC9234325

[acel70282-bib-0072] Jamerson, T. A. , and C. Aguh . 2021. “An Approach to Patients With Alopecia.” Medical Clinics of North America 105, no. 4: 599–610. 34059240 10.1016/j.mcna.2021.04.002

[acel70282-bib-0074] Jin, Z. , B. Du , X. Jiao , et al. 2025. “Decoding Sexually Dimorphic Proteomic Landscapes in the Context of Aging and Mortality.” Communications Medicine 5, no. 1: 403. 10.1038/s43856-025-01113-0. 41023390 PMC12479788

[acel70282-bib-0075] Jumper, J. , R. Evans , A. Pritzel , et al. 2021. “Highly Accurate Protein Structure Prediction With AlphaFold.” Nature 596, no. 7873: 583–589. 10.1038/s41586-021-03819-2. 34265844 PMC8371605

[acel70282-bib-0076] Jun, L. , M. Robinson , T. Geetha , T. L. Broderick , and J. R. Babu . 2023. “Prevalence and Mechanisms of Skeletal Muscle Atrophy in Metabolic Conditions.” International Journal of Molecular Sciences 24, no. 3: 2973. 36769296 10.3390/ijms24032973 PMC9917738

[acel70282-bib-0077] Kaatsch, H. L. , L. Kubitscheck , S. Wagner , et al. 2025. “Routine CT Diagnostics Cause Dose‐Dependent Gene Expression Changes in Peripheral Blood Cells.” International Journal of Molecular Sciences 26, no. 7: 3185. 10.3390/ijms26073185. 40243988 PMC11989232

[acel70282-bib-0078] Kaltschmidt, C. , J. F. Greiner , and B. Kaltschmidt . 2021. “The Transcription Factor NF‐κB in Stem Cells and Development.” Cells 10, no. 8: 2042. 34440811 10.3390/cells10082042 PMC8391683

[acel70282-bib-0079] Kanoni, S. , S. E. Graham , Y. Wang , et al. 2022. “Implicating Genes, Pleiotropy, and Sexual Dimorphism at Blood Lipid Loci Through Multi‐Ancestry Meta‐Analysis.” Genome Biology 23, no. 1: 268. 36575460 10.1186/s13059-022-02837-1 PMC9793579

[acel70282-bib-0080] Katthika, V. K. , and E. I. Auerkari . 2018. Ectodermal Dysplasia. In 11th International Dentistry Scientific Meeting (IDSM 2017), 230–238. Atlantis Press.

[acel70282-bib-0081] Kciuk, M. , A. Gielecińska , A. Budzinska , M. Mojzych , and R. Kontek . 2022. “Metastasis and MAPK Pathways.” International Journal of Molecular Sciences 23, no. 7: 3847. 35409206 10.3390/ijms23073847 PMC8998814

[acel70282-bib-0083] Kim, M. S. , S. Khurshid , S. Kany , et al. 2025. “Machine Learning‐Based Plasma Protein Risk Score Improves Atrial Fibrillation Prediction Over Clinical and Genomic Models.” Circulation: Genomic and Precision Medicine 18: e004943. 10.1161/CIRCGEN.124.004943. 40525300 PMC12257488

[acel70282-bib-0198] Kinde, M. Z. , T. A. Mekuria , A. T. Gessese , and B. A. Mengistu . 2024. “Molecular Mechanisms of Hair Follicle Development.” Scientific World Journal 2024: 5259055. 10.1155/tswj/5259055. 39628556 PMC11614512

[acel70282-bib-0084] Kiss, N. , C. M. Prado , A. R. Curtis , et al. 2024. “Risk Factors for Low Muscle Mass, Malnutrition, and (Probable‐) Sarcopenia in Adults With or Without a History of Cancer in the UK Biobank.” Clinical Nutrition 43, no. 7: 1736–1746. 10.1016/j.clnu.2024.05.041. 38843582

[acel70282-bib-0085] Koupenova, M. , L. Clancy , H. A. Corkrey , and J. E. Freedman . 2018. “Circulating Platelets as Mediators of Immunity, Inflammation, and Thrombosis.” Circulation Research 122, no. 2: 337–351. 10.1161/CIRCRESAHA.117.310795. 29348254 PMC5777300

[acel70282-bib-0086] Kuan, P. F. , S. Clouston , X. Yang , R. Kotov , E. Bromet , and B. J. Luft . 2020. “Molecular Linkage Between Post‐Traumatic Stress Disorder and Cognitive Impairment: A Targeted Proteomics Study of World Trade Center Responders.” Translational Psychiatry 10, no. 1: 269. 32753605 10.1038/s41398-020-00958-4 PMC7403297

[acel70282-bib-0087] Kuo, C. L. , P. Liu , G. Drouard , et al. 2025. “A Proteomic Signature of Healthspan.” Proceedings of the National Academy of Sciences of the United States of America 122, no. 23: e2414086122. 10.1073/pnas.2414086122. 40478878 PMC12168021

[acel70282-bib-0088] Kuwabara, Y. , T. Yokokawa , S. E. Lemay , et al. 2025. “Exploratory Study of Prognostic Plasma Biomarkers in Patients With Pulmonary Arterial Hypertension.” American Journal of Pathology 195: 1376–1393. 10.1016/j.ajpath.2025.04.018. 40451321 PMC12405927

[acel70282-bib-0089] Kwack, M. H. , O. B. Hamida , W. J. Lee , and M. K. Kim . 2024. “EDA‐A2 Increases Lipid Production in EDA2R‐Expressing Human Sebocytes.” Journal of Dermatological Science 113, no. 1: 34–37. 38030512 10.1016/j.jdermsci.2023.11.005

[acel70282-bib-0199] Kwack, M. H. , J. C. Kim , and M. K. Kim . 2019. “Ectodysplasin‐A2 Induces Apoptosis in Cultured Human Hair Follicle Cells and Promotes Regression of Hair Follicles in Mice.” Biochemical and Biophysical Research Communications 520, no. 2: 428–433. 10.1016/j.bbrc.2019.10.031. 31607478

[acel70282-bib-0090] Lan, X. , V. Kumar , A. Jha , et al. 2020. “EDA2R Mediates Podocyte Injury in High Glucose Milieu.” Biochimie 174: 74–83. 32304771 10.1016/j.biochi.2020.04.003 PMC7282945

[acel70282-bib-0091] Larsson, S. C. , and S. Burgess . 2021. “Causal Role of High Body Mass Index in Multiple Chronic Diseases: A Systematic Review and Meta‐Analysis of Mendelian Randomization Studies.” BMC Medicine 19, no. 1: 320. 10.1186/s12916-021-02188-x. 34906131 PMC8672504

[acel70282-bib-0092] Lee, C. W. , B. Y. H. Wang , S. H. Wong , et al. 2025. “Ginkgolide B Increases Healthspan and Lifespan of Female Mice.” Nature Aging 5, no. 2: 237–258. 39890935 10.1038/s43587-024-00802-0

[acel70282-bib-0093] Lee, J. , and T. Tumbar . 2012. “Hairy Tale of Signaling in Hair Follicle Development and Cycling.” Seminars in Cell & Developmental Biology 23, no. 8: 906–916. 22939761 10.1016/j.semcdb.2012.08.003 PMC3496046

[acel70282-bib-0095] Liang, A. , Y. Fang , L. Ye , et al. 2023. “Signaling Pathways in Hair Aging.” Frontiers in Cell and Developmental Biology 11: 1278278. 38033857 10.3389/fcell.2023.1278278 PMC10687558

[acel70282-bib-0096] Lim, H. W. , H. J. Kim , C. Y. Jeon , et al. 2024. “Hair Growth Promoting Effects of 15‐Hydroxyprostaglandin Dehydrogenase Inhibitor in Human Follicle Dermal Papilla Cells.” International Journal of Molecular Sciences 25, no. 13: 7485. 39000592 10.3390/ijms25137485 PMC11242524

[acel70282-bib-0097] Lind, L. , M. Mazidi , R. Clarke , D. A. Bennett , and R. Zheng . 2024. “Measured and Genetically Predicted Protein Levels and Cardiovascular Diseases in UK Biobank and China Kadoorie Biobank.” Nature Cardiovascular Research 3, no. 10: 1189–1198. 10.1038/s44161-024-00545-6 PMC11473359 39322770

[acel70282-bib-0098] Llaurador‐Coll, M. , S. Rios , J. F. García‐Gavilán , N. Babio , E. Vilella , and J. Salas‐Salvadó . 2023. “Plasma Levels of Neurology‐Related Proteins Are Associated With Cognitive Performance in an Older Population With Overweight/Obesity and Metabolic Syndrome.” GeroScience 45, no. 4: 2457–2470. 36964401 10.1007/s11357-023-00764-y PMC10651568

[acel70282-bib-0099] López‐Otín, C. , M. A. Blasco , L. Partridge , M. Serrano , and G. Kroemer . 2023. “Hallmarks of Aging: An Expanding Universe.” Cell 186, no. 19: 243–278. 10.1016/j.cell.2022.11.001. 36599349

[acel70282-bib-0101] Lund, C. , P. Ranea‐Robles , S. Falk , et al. 2024. “Protection Against Overfeeding‐Induced Weight Gain Is Preserved in Obesity but Does Not Require FGF21 or MC4R.” Nature Communications 15, no. 1: 1192. 10.1038/s41467-024-45223-0. PMC10853283 38331907

[acel70282-bib-0102] Ma, L. Z. , W. S. Liu , Y. He , et al. 2025. “Plasma Proteomics Identify Novel Biomarkers and Dynamic Patterns of Biological Aging.” Journal of Advanced Research S2090‐1232, no. 25: 00297–8. 10.1016/j.jare.2025.05.004.40328427

[acel70282-bib-0103] Maldonado, E. , S. Morales‐Pison , F. Urbina , and A. Solari . 2023. “Aging Hallmarks and the Role of Oxidative Stress.” Antioxidants 12, no. 6: 651. 10.3390/antiox12030651. 36978899 PMC10044767

[acel70282-bib-0104] Malhotra, K. , and B. Madke . 2023. “An Updated Review on Current Treatment of Alopecia Areata and Newer Therapeutic Options.” International Journal of Trichology 15: 3–12. 37305188 10.4103/ijt.ijt_28_21 PMC10251289

[acel70282-bib-0105] Margara‐Escudero, H. J. , I. Paz‐Graniel , J. F. García‐Gavilán , et al. 2025. “Plasma Neurology‐Related Proteins Associated With Cognition Are Modulated by Lifestyle in Adults.” eBioMedicine 120: 105933. 10.1016/j.ebiom.2025.105933. 40972225 PMC12478270

[acel70282-bib-0106] Mariani, V. , M. Biasini , A. Barbato , and T. Schwede . 2013. “lDDT: A Local Superposition‐Free Score for Comparing Protein Structures and Models Using Distance Difference Tests.” Bioinformatics 29, no. 21: 2722–2728. 23986568 10.1093/bioinformatics/btt473 PMC3799472

[acel70282-bib-0107] Mariean, C. R. , O. M. Tiucă , A. Mariean , and O. S. Cotoi . 2023. “Cancer Cachexia: New Insights and Future Directions.” Cancers 15, no. 23: 5590. 38067294 10.3390/cancers15235590 PMC10705516

[acel70282-bib-0108] Mercuri, E. , C. G. Bönnemann , and F. Muntoni . 2019. “Muscular Dystrophies.” Lancet 394, no. 10213: 2025–2038. 10.1016/S0140-6736(19)32910-1. 31789220

[acel70282-bib-0109] Miclea, A. , J. Zurawski , B. C. Healy , et al. 2025. “Novel Serum Biomarker Associations With 7 Tesla MRI‐Defined Cortical Lesions, Leptomeningeal Enhancement, and Deep Gray Matter Volume in Early Multiple Sclerosis.” Scientific Reports 15, no. 1: 12032. 40200016 10.1038/s41598-025-95229-x PMC11978968

[acel70282-bib-0110] Mikkola, M. L. 2008. “TNF Superfamily in Skin Appendage Development.” Cytokine & Growth Factor Reviews 19, no. 3–4: 219–230. 18495521 10.1016/j.cytogfr.2008.04.008

[acel70282-bib-0112] Moneva‐Sakelarieva, M. , Y. Kobakova , S. Konstantinov , et al. 2025. “The Role of the Transcription Factor NF‐kB in the Pathogenesis of Inflammation and Carcinogenesis. Modulation Capabilities.” Pharmacia 72: 1–13.

[acel70282-bib-0113] Moore, T. M. , S. Lee , T. Olsen , et al. 2023. “Conserved Multi‐Tissue Transcriptomic Adaptations to Exercise Training in Humans and Mice.” Cell Reports 42, no. 5: 112499. 10.1016/j.celrep.2023.112499. 37178122 PMC11352395

[acel70282-bib-0114] Moreira‐Pais, A. , R. Ferreira , P. A. Oliveira , and J. A. Duarte . 2021. “Sarcopenia Versus Cancer Cachexia: The Muscle Wasting Continuum in Healthy and Diseased Aging.” Biogerontology 22, no. 5: 459–477. 34324116 10.1007/s10522-021-09932-z

[acel70282-bib-0115] Mörseburg, A. , Y. Zhao , K. A. Kentistou , J. R. B. Perry , K. K. Ong , and F. R. Day . 2025. “Genetic Determinants of Proteomic Aging.” NPJ Aging 11, no. 1: 30. 10.1038/s41514-025-00205-4. 40287427 PMC12033249

[acel70282-bib-0117] Nicu, C. , J. Jackson , A. Shahmalak , J. Pople , D. Ansell , and R. Paus . 2023. “Adiponectin Negatively Regulates Pigmentation, Wnt/β‐Catenin and HGF/c‐Met Signalling Within Human Scalp Hair Follicles Ex Vivo.” Archives of Dermatological Research 315, no. 3: 603–612. 34854998 10.1007/s00403-021-02291-2

[acel70282-bib-0118] Nielsen, R. L. , T. Monfeuga , R. R. Kitchen , et al. 2024. “Data‐Driven Identification of Predictive Risk Biomarkers for Subgroups of Osteoarthritis Using Interpretable Machine Learning.” Nature Communications 15, no. 1: 2817. 10.1038/s41467-024-46663-4. PMC10985086 38561399

[acel70282-bib-0119] Ntshingila, S. , O. Oputu , A. T. Arowolo , and N. P. Khumalo . 2023. “Androgenetic Alopecia: An Update.” JAAD International 13: 150–158. 37823040 10.1016/j.jdin.2023.07.005 PMC10562178

[acel70282-bib-0120] Oh, H. S. , Y. Le Guen , N. Rappoport , et al. 2025. “Plasma Proteomics Links Brain and Immune System Aging With Healthspan and Longevity.” Nature Medicine 31, no. 8: 2703–2711. 10.1038/s41591-025-03798-1. PMC12353788 40634782

[acel70282-bib-0121] Oiwoh, S. O. , A. O. Enitan , O. T. Adegbosin , A. O. Akinboro , and E. O. Onayemi . 2024. “Androgenetic Alopecia: A Review.” Nigerian Postgraduate Medical Journal 31, no. 2: 85–92. 38826011 10.4103/npmj.npmj_47_24

[acel70282-bib-0122] Open Targets Platform . Accessed on 17 June 2025. https://platform.opentargets.org/. 2025.

[acel70282-bib-0123] Ottens, F. , A. Franz , and T. Hoppe . 2021. “Build‐UPS and Break‐Downs: Metabolism Impacts on Proteostasis and Aging.” Cell Death and Differentiation 28, no. 2: 505–521. 33398091 10.1038/s41418-020-00682-y PMC7862225

[acel70282-bib-0124] Özen, S. D. , and S. Kir . 2024. “Ectodysplasin A2 Receptor Signaling in Skeletal Muscle Pathophysiology.” Trends in Molecular Medicine 30, no. 5: 471–483. 38443222 10.1016/j.molmed.2024.02.002

[acel70282-bib-0125] Papier, K. , J. R. Atkins , T. Y. N. Tong , et al. 2024. “Identifying Proteomic Risk Factors for Cancer Using Prospective and Exome Analyses of 1,463 Circulating Proteins and Risk of 19 Cancers in the UK Biobank.” Nature Communications 15, no. 1: 4010. 10.1038/s41467-024-48017-6. PMC11096312 38750076

[acel70282-bib-0200] Papukashvili, D. , N. Rcheulishvili , C. Liu , et al. 2021. “Perspectives on miRNAs Targeting DKK1 for Developing Hair Regeneration Therapy.” Cells 10, no. 11. 10.3390/cells10112957. PMC8616136 34831180

[acel70282-bib-0126] Patel, H. , N. J. Ashton , R. J. B. Dobson , et al. 2021. “Proteomic Blood Profiling in Mild, Severe and Critical COVID‐19 Patients.” Scientific Reports 11, no. 1: 6357. 10.1038/s41598-021-85877-0. 33737684 PMC7973581

[acel70282-bib-0127] Perez, K. , S. Ciotlos , J. McGirr , et al. 2022. “Single Nuclei Profiling Identifies Cell Specific Markers of Skeletal Muscle Aging, Frailty, and Senescence.” Aging 14, no. 23: 9393–9422. 36516485 10.18632/aging.204435 PMC9792217

[acel70282-bib-0128] Perez, S. M. , B. Nguyen , and M. M. Senna . 2025. “Efficacy and Safety of Bicalutamide in Female Hair Loss: A Review of the Literature.” JAAD Reviews v4: 63–68.

[acel70282-bib-0129] Phillips, T. G. , W. P. Slomiany , and R. Allison . 2017. “Hair Loss: Common Causes and Treatment.” American Family Physician 96: 371–378. 28925637

[acel70282-bib-0130] Pietzner, M. , B. Uluvar , K. J. Kolnes , et al. 2024. “Systemic Proteome Adaptions to 7‐Day Complete Caloric Restriction in Humans.” Nature Metabolism 6, no. 4: 764–777. 10.1038/s42255-024-01008-9 PMC7617311 38429390

[acel70282-bib-0131] Piraccini, B. M. , U. Blume‐Peytavi , F. Scarci , et al. 2022. “Efficacy and Safety of Topical Finasteride Spray Solution for Male Androgenetic Alopecia: A Phase III, Randomized, Controlled Clinical Trial.” Journal of the European Academy of Dermatology and Venereology 36, no. 2: 286–294. 34634163 10.1111/jdv.17738 PMC9297965

[acel70282-bib-0132] Pirastu, N. , P. K. Joshi , P. S. de Vries , et al. 2017. “GWAS for Male‐Pattern Baldness Identifies 71 Susceptibility Loci Explaining 38% of the Risk.” Nature Communications 8, no. 1: 1584. 10.1038/s41467-017-01490-8. PMC5691155 29146897

[acel70282-bib-0133] Pozo‐Pérez, L. , P. Tornero‐Esteban , and E. López‐Bran . 2024. “Clinical and Preclinical Approach in AGA Treatment: A Review of Current and New Therapies in the Regenerative Field.” Stem Cell Research & Therapy 15, no. 1: 260. 39148125 10.1186/s13287-024-03801-5 PMC11328498

[acel70282-bib-0134] Pratt, C. H. , L. E. King Jr. , A. G. Messenger , A. M. Christiano , and J. P. Sundberg . 2017. “Alopecia Areata.” Nature Reviews Disease Primers 3: 17011. 10.1038/nrdp.2017.11 PMC5573125 28300084

[acel70282-bib-0136] Prescott, J. A. , J. P. Mitchell , and S. J. Cook . 2021. “Inhibitory Feedback Control of NF‐κB Signalling in Health and Disease.” Biochemical Journal 478, no. 13: 2619–2664. 34269817 10.1042/BCJ20210139 PMC8286839

[acel70282-bib-0137] Prodi, D. A. , N. Pirastu , G. Maninchedda , et al. 2008. “EDA2R Is Associated With Androgenetic Alopecia.” Journal of Investigative Dermatology 128, no. 9: 2268–2270. 18385763 10.1038/jid.2008.60

[acel70282-bib-0138] Qian, H. , C. Wu , B. Li , A. Rosenzweig , and M. Wang . 2025. “Plasma Proteomics Linking Primary and Secondary Diseases: Insights Into Molecular Mediation From UK Biobank Data. *Medrxiv*.” 10.1101/2025.08.29.25334726. 42551443

[acel70282-bib-0140] Redler, S. , F. F. Brockschmidt , R. Tazi‐Ahnini , et al. 2012. “Investigation of the Male Pattern Baldness Major Genetic Susceptibility Loci AR/EDA2R and 20p11 in Female Pattern Hair Loss.” British Journal of Dermatology 166, no. 6: 1314–1318. 22309448 10.1111/j.1365-2133.2012.10877.x

[acel70282-bib-0141] Rietmann, S. J. , N. Cochet‐Faivre , H. Dropsy , V. Jagannathan , L. Chevallier , and T. Leeb . 2024. “EDA Missense Variant in a Cat With X‐Linked Hypohidrotic Ectodermal Dysplasia.” Genes 15, no. 7: 854. 39062633 10.3390/genes15070854 PMC11276485

[acel70282-bib-0142] Roger, L. , F. Tomas , and V. Gire . 2021. “Mechanisms and Regulation of Cellular Senescence.” International Journal of Molecular Sciences 22, no. 23: 13173. 34884978 10.3390/ijms222313173 PMC8658264

[acel70282-bib-0201] Ryu, Y. , C. Kim , Y. R. Park , et al. 2022. “Wnt/Beta‐Catenin Signaling Activator Restores Hair Regeneration Suppressed by Diabetes Mellitus.” BMB Rep 55, no. 11: 559–564. . 10.5483/BMBRep.2022.55.11.081. 36016500 PMC9712708

[acel70282-bib-0143] Sadasivam, I. P. , R. Sambandam , D. Kaliyaperumal , and J. E. Dileep . 2024. “Androgenetic Alopecia in Men: An Update on Genetics.” Indian Journal of Dermatology 69, no. 3: 282. 10.4103/ijd.ijd_729_23 PMC11305502 39119311

[acel70282-bib-0144] Safiri, S. , K. M. Asghari , and M. J. Sullman . 2023. “The Global Burden of Diseases and Injuries Among Older Adults.” Science 20: 21.

[acel70282-bib-0147] Schiaffino, S. , C. Reggiani , T. Akimoto , and B. Blaauw . 2021. “Molecular Mechanisms of Skeletal Muscle Hypertrophy.” Journal of Neuromuscular Diseases 8, no. 2: 169–183. 10.3233/JND-200568. 33216041 PMC8075408

[acel70282-bib-0148] Schneider, M. R. , R. Schmidt‐Ullrich , and R. Paus . 2009. “The Hair Follicle as a Dynamic Miniorgan.” Current Biology 19: R132–R142. 10.1016/j.cub.2008.12.005. 19211055

[acel70282-bib-0149] Semenova, E. A. , E. Pranckevičienė , E. A. Bondareva , L. J. Gabdrakhmanova , and I. I. Ahmetov . 2023. “Identification and Characterization of Genomic Predictors of Sarcopenia and Sarcopenic Obesity Using UK Biobank Data.” Nutrients 15, no. 3: 758. 10.3390/nu15030758. 36771461 PMC9920138

[acel70282-bib-0150] Sinha, S. K. , S. Zachariah , H. I. Quiñones , M. Shindo , and P. M. Chaudhary . 2002. “Role of TRAF3 and ‐6 in the Activation of the NF‐κB and JNK Pathways by X‐Linked Ectodermal Dysplasia Receptor.” Journal of Biological Chemistry 277, no. 47: 44953–44961. 12270937 10.1074/jbc.M207923200

[acel70282-bib-0151] Sproston, N. R. , and J. J. Ashworth . 2018. “Role of C‐Reactive Protein at Sites of Inflammation and Infection.” Frontiers in Immunology 9: 754. 10.3389/fimmu.2018.00754. 29706967 PMC5908901

[acel70282-bib-0152] Stefanato, C. M. 2010. “Histopathology of Alopecia: A Clinicopathological Approach to Diagnosis.” Histopathology 56: 24–38. 20055903 10.1111/j.1365-2559.2009.03439.x

[acel70282-bib-0153] Sun, S. C. 2017. “The Non‐Canonical NF‐κB Pathway in Immunity and Inflammation.” Nature Reviews Immunology 17, no. 9: 545–558. 10.1038/nri.2017.52 PMC5753586 28580957

[acel70282-bib-0154] Svensson‐Färbom, P. , M. Ohlson Andersson , P. Almgren , et al. 2014. “Cystatin C Identifies Cardiovascular Risk Better Than Creatinine‐Based Estimates of Glomerular Filtration in Middle‐Aged Individuals Without a History of Cardiovascular Disease.” Journal of Internal Medicine 275, no. 5: 506–521. 10.1111/joim.12169. 24279862

[acel70282-bib-0155] Tang, X. , C. Cao , Y. Liang , et al. 2023. “Adipose‐Derived Stem Cell Exosomes Antagonize the Inhibitory Effect of Dihydrotestosterone on Hair Follicle Growth by Activating Wnt/*β*‐Catenin Pathway.” Stem Cells International 2023: 5548112. 10.1155/2023/5548112. 37810630 PMC10551537

[acel70282-bib-0156] Taylor, D. F. , E. G. Reisman , A. Garnham , et al. 2025. “Exercise Induces Time‐Dependent but Not Sex‐Specific Transcriptomic Changes in Healthy Human Skeletal Muscle. *bioRxiv*.” 10.1101/2025.08.17.667354.

[acel70282-bib-0158] Tian, T. , M. Liu , P. J. Little , H. Strijdom , J. Weng , and S. Xu . 2025. “Emerging Roles of GDF15 in Metabolic and Cardiovascular Diseases.” Research 8: 0832. 10.34133/research.0832. 40837873 PMC12361751

[acel70282-bib-0162] Vadaq, N. , Y. Zhang , W. A. Vos , et al. 2023. “High‐Throughput Proteomic Analysis Reveals Systemic Dysregulation in Virally Suppressed People Living With HIV.” JCI Insight 8, no. 11: e166166. 37079385 10.1172/jci.insight.166166 PMC10393229

[acel70282-bib-0163] Vanderwolf, K. , C. Kyle , and C. Davy . 2023. “A Review of Sebum in Mammals in Relation to Skin Diseases, Skin Function, and the Skin Microbiome.” PeerJ 11: e16680. 38144187 10.7717/peerj.16680 PMC10740688

[acel70282-bib-0164] Varadi, M. , D. Bertoni , P. Magana , et al. 2024. “AlphaFold Protein Structure Database in 2024: Providing Structure Coverage for Over 214 Million Protein Sequences.” Nucleic Acids Research 52, no. D1: D368–D375. 10.1093/nar/gkad1011. 37933859 PMC10767828

[acel70282-bib-0165] Varothai, S. , and W. F. Bergfeld . 2014. “Androgenetic Alopecia: An Evidence‐Based Treatment Update.” American Journal of Clinical Dermatology 15, no. 3: 217–230. 10.1007/s40257-014-0077-5. 24848508

[acel70282-bib-0166] Von Haehling, S. , N. Ebner , M. R. Dos Santos , J. Springer , and S. D. Anker . 2017. “Muscle Wasting and Cachexia in Heart Failure: Mechanisms and Therapies.” Nature Reviews Cardiology 14, no. 6: 323–341. 28436486 10.1038/nrcardio.2017.51

[acel70282-bib-0167] von Renesse, J. , and P. Mirtschink . 2023. “Ectodysplasin A2 Receptor‐NF‐κB‐Inducing Kinase Axis: A New Player in Muscle Wasting to Cancer Cachexia.” Signal Transduction and Targeted Therapy 8, no. 1: 383. 37807033 10.1038/s41392-023-01617-y PMC10560659

[acel70282-bib-0168] Wagemann, O. , G. Nübling , F. J. Martínez‐Murcia , et al. 2025. “Exploratory Analysis of the Proteomic Profile in Plasma in Adults With Down Syndrome in the Context of Alzheimer's Disease.” Alzheimer's & Dementia 21, no. 3: e70040. 10.1002/alz.70040 PMC11923571 40110647

[acel70282-bib-0170] Wang, B. , A. Pozarickij , M. Mazidi , et al. 2025. “Comparative Studies of 2168 Plasma Proteins Measured by Two Affinity‐Based Platforms in 4000 Chinese Adults.” Nature Communications 16, no. 1: 1869. 10.1038/s41467-025-56935-2 PMC11845630 39984443

[acel70282-bib-0171] Wang, K. , H. Liu , Q. Hu , et al. 2022. “Epigenetic Regulation of Aging: Implications for Interventions of Aging and Diseases.” Signal Transduction and Targeted Therapy 7, no. 1: 374. 36336680 10.1038/s41392-022-01211-8 PMC9637765

[acel70282-bib-0172] Wang, R. , T. Zhong , Q. Bian , et al. 2023. “PROTAC Degraders of Androgen Receptor‐Integrated Dissolving Microneedles for Androgenetic Alopecia and Recrudescence Treatment via Single Topical Administration.” Small Methods 7, no. 1: 2201293. 10.1002/smtd.202201293 36538748

[acel70282-bib-0173] Wen, J. 2025. “Refining the Generation, Interpretation and Application of Multi‐Organ, Multi‐Omics Biological Aging Clocks.” Nature Aging 5, no. 5: 1897–1913. 10.1038/s43587-025-00928-9. 40764431

[acel70282-bib-0174] Wiedmer, P. , T. Jung , J. P. Castro , et al. 2021. “Sarcopenia—Molecular Mechanisms and Open Questions.” Ageing Research Reviews 65: 101200. 10.1016/j.arr.2020.101200. 33130247

[acel70282-bib-0175] Wiens, G. D. , and G. W. Glenney . 2011. “Origin and Evolution of TNF and TNF Receptor Superfamilies.” Developmental & Comparative Immunology 35, no. 12: 1324–1335. 21527275 10.1016/j.dci.2011.03.031

[acel70282-bib-0176] Willeit, P. , L. Tschiderer , E. Allara , et al. 2020. “Carotid Intima‐Media Thickness Progression as Surrogate Marker for Cardiovascular Risk: Meta‐Analysis of 119 Clinical Trials Involving 100,667 Patients.” Circulation 142, no. 7: 621–642. 10.1161/CIRCULATIONAHA.120.046361. 32546049 PMC7115957

[acel70282-bib-0202] Williams, R. , G. E. Westgate , A. D. Pawlus , S. K. Sikkink , and M. J. Thornton . 2021. “Age‐Related Changes in Female Scalp Dermal Sheath and Dermal Fibroblasts: How the Hair Follicle Environment Impacts Hair Aging.” Journal of Investigative Dermatology 141, no. 4S: 1041–1051. 10.1016/j.jid.2020.11.009. 33326808

[acel70282-bib-0177] Wisniewski, S. A. , and W. H. Trzeciak . 2012. “A New Mutation Resulting in the Truncation of the TRAF6‐Interacting Domain of XEDAR: A Possible Novel Cause of Hypohidrotic Ectodermal Dysplasia.” Journal of Medical Genetics 49, no. 8: 499–501. 22889853 10.1136/jmedgenet-2012-100877

[acel70282-bib-0178] Wu, N. , S. Wang , Y. Zhang , and S. Wang . 2025. “Research Progress on Anti‐Inflammatory Mechanism of Inula Cappa.” International Journal of Molecular Sciences 26, no. 5: 1911. 40076538 10.3390/ijms26051911 PMC11900443

[acel70282-bib-0179] Xiao, Y. , Y. Zhang , S. Deng , X. Yang , and X. Yao . 2025. “Immune and Non‐Immune Interactions in the Pathogenesis of Androgenetic Alopecia.” Clinical Reviews in Allergy & Immunology 68, no. 1: 22. 40024940 10.1007/s12016-025-09034-5

[acel70282-bib-0180] Yan, M. , L. C. Wang , S. G. Hymowitz , et al. 2000. “Two‐Amino Acid Molecular Switch in an Epithelial Morphogen That Regulates Binding to Two Distinct Receptors.” Science 290, no. 5491: 523–527. 10.1126/science.290.5491.523. 11039935

[acel70282-bib-0181] Yang, D. Z. , J. Kua , and W. S. Lim . 2025. “The Impact of Lifestyle Factors Across the Life Course on Sarcopenia and Physical Frailty.” Current Opinion in Clinical Nutrition and Metabolic Care 28, no. 3: 208–223. 10.1097/MCO.0000000000001111. 39907147

[acel70282-bib-0182] Yang, R. , Y. Mei , Y. Jiang , et al. 2022. “Ectodysplasin A (EDA) Signaling: From Skin Appendage to Multiple Diseases.” International Journal of Molecular Sciences 23, no. 16: 8911. 36012178 10.3390/ijms23168911 PMC9408960

[acel70282-bib-0183] Yang, W. , S. Jin , J. Jiang , W. J. Ji , and Q. He . 2025. “A Novel Missense Mutation at EDA2R Gene Identified in a Case Study Associated With Hypohidrotic Ectodermal Dysplasia.” Regenerative Medicine and Dentistry 2: 2.

[acel70282-bib-0184] You, J. , Y. Guo , Y. Zhang , et al. 2023. “Plasma Proteomic Profiles Predict Individual Future Health Risk.” Nature Communications 14, no. 1: 7817. 10.1038/s41467-023-43575-7. PMC10684756 38016990

[acel70282-bib-0185] Yu, K. , C. Huang , F. Wan , et al. 2023. “Structural Insights Into Pathogenic Mechanism of Hypohidrotic Ectodermal Dysplasia Caused by Ectodysplasin A Variants.” Nature Communications 14, no. 1: 767. 10.1038/s41467-023-36367-6 PMC9918506 36765055

[acel70282-bib-0186] Zaky, M. S. , H. Abo Khodeir , H. A. Ahmed , and M. L. Elsaie . 2021. “Therapeutic Implications of Topical Cetirizine 1% in Treatment of Male Androgenetic Alopecia: A Case‐Controlled Study.” Journal of Cosmetic Dermatology 20, no. 4: 1154–1159. 10.1111/jocd.13940. 33417284

[acel70282-bib-0187] Zemla, A. 2003. “LGA: A Method for Finding 3D Similarities in Protein Structures.” Nucleic Acids Research 31, no. 13: 3370–3374. 12824330 10.1093/nar/gkg571 PMC168977

[acel70282-bib-0188] Zhang, K. , Y. Ma , Y. Luo , et al. 2023. “Metabolic Diseases and Healthy Aging: Identifying Environmental and Behavioral Risk Factors and Promoting Public Health.” Frontiers in Public Health 11: 1253506. 37900047 10.3389/fpubh.2023.1253506 PMC10603303

[acel70282-bib-0189] Zhang, L. , L. E. Pitcher , M. J. Yousefzadeh , L. J. Niedernhofer , P. D. Robbins , and Y. Zhu . 2022. “Cellular Senescence: A Key Therapeutic Target in Aging and Diseases.” Journal of Clinical Investigation 132, no. 15: e158450. 35912854 10.1172/JCI158450 PMC9337830

[acel70282-bib-0190] Zhang, Y. , Y. Guo , Y. He , et al. 2025. “Large‐Scale Proteomic Analyses of Incident Alzheimer's Disease Reveal New Pathophysiological Insights and Potential Therapeutic Targets.” Molecular Psychiatry 30, no. 6: 2347–2361. 39562718 10.1038/s41380-024-02840-x

[acel70282-bib-0192] Zhang, Y. X. , M. Y. Ou , Z. H. Yang , Y. Sun , Q. F. Li , and S. B. Zhou . 2023. “Adipose Tissue Aging Is Regulated by an Altered Immune System.” Frontiers in Immunology 14: 1125395. 36875140 10.3389/fimmu.2023.1125395 PMC9981968

[acel70282-bib-0191] Zhang, Y. , and J. Skolnick . 2004. “Scoring Function for Automated Assessment of Protein Structure Template Quality.” Proteins: Structure, Function, and Bioinformatics 57, no. 4: 702–710. 10.1002/prot.20264 15476259

[acel70282-bib-0193] Zhou, X. , Y. Jiao , W. Zhang , and W. Li . 2022. “Androgens/Androgen Receptor in the Management of Skin Diseases.” Journal of Biosciences and Medicines 10, no. 12: 180–200.

[acel70282-bib-0194] Zoodsma, M. , C. Beuchel , S. Yasmeen , et al. 2025. “A Genetic Map of Human Metabolism Across the Allele Frequency Spectrum.” Nature Genetics 57: 2445–2455. 10.1038/s41588-025-02355-3. 41044249 PMC12513840

